# Long-term fixation impact on archived human nervous tissues for sequencing-based transcriptomics

**DOI:** 10.1093/braincomms/fcaf428

**Published:** 2025-10-30

**Authors:** Stephanie Howe, Heather McCann, Zherui Xiong, Quan Nguyen, Frederik J Steyn, Shyuan T Ngo, Jeryn Chang

**Affiliations:** Australian Institute of Bioengineering and Nanotechnology, The University of Queensland, Brisbane, QLD 4072, Australia; Neuroscience Research Australia, Randwick, NSW 2031, Australia; Queensland Institute of Medical Research, Herston, QLD 4006, Australia; Queensland Institute of Medical Research, Herston, QLD 4006, Australia; School of Biomedical Sciences, The University of Queensland, Brisbane, QLD 4072, Australia; Australian Institute of Bioengineering and Nanotechnology, The University of Queensland, Brisbane, QLD 4072, Australia; School of Biomedical Sciences, The University of Queensland, Brisbane, QLD 4072, Australia

**Keywords:** brain bank, FFPE, tissue fixation, RNA, transcriptomics

## Abstract

The advent of transcriptomics technologies compatible with formalin-fixed paraffin-embedded tissues has enabled greater opportunities for scientists to interrogate the transcriptome of archived tissues, especially those from rare neurodegenerative diseases. Tissues from brain banks are often placed in fixative for weeks or years due to limitations in resources and storage space. We investigated the impact of long-term fixation for sequencing-based transcriptomics in human formalin-fixed paraffin-embedded lumbar spinal cord and hypothalamic tissues sourced from the Sydney Brain Bank. Extended fixation times were not associated with RNA quality metrics, RNA integrity number or DV200. However, fixation duration was associated with poorer ligation of transcriptome probes, leading to poorer detection of RNA molecules. This led to lower measured central nervous system gene expression, severely impacting the interpretability of the transcriptome. The findings of this study underscore the importance of adaptability and standardization among brain banks, especially in the face of rapid technological advancements. There is a need to foster collaborative efforts between researchers, technology providers and brain banks worldwide to ensure tissue compatibility with emerging technologies.

## Teaser

Howe *et al.* report that extended formalin fixation of central nervous system tissues hinders gene expression analysis and that common RNA quality metrics do not reliably indicate archived sample quality. Collaboration among researchers, brain banks and industry is essential to ensure tissue compatibility as new technologies emerge.

## Graphical Abstract

**Graphical Abstract:**
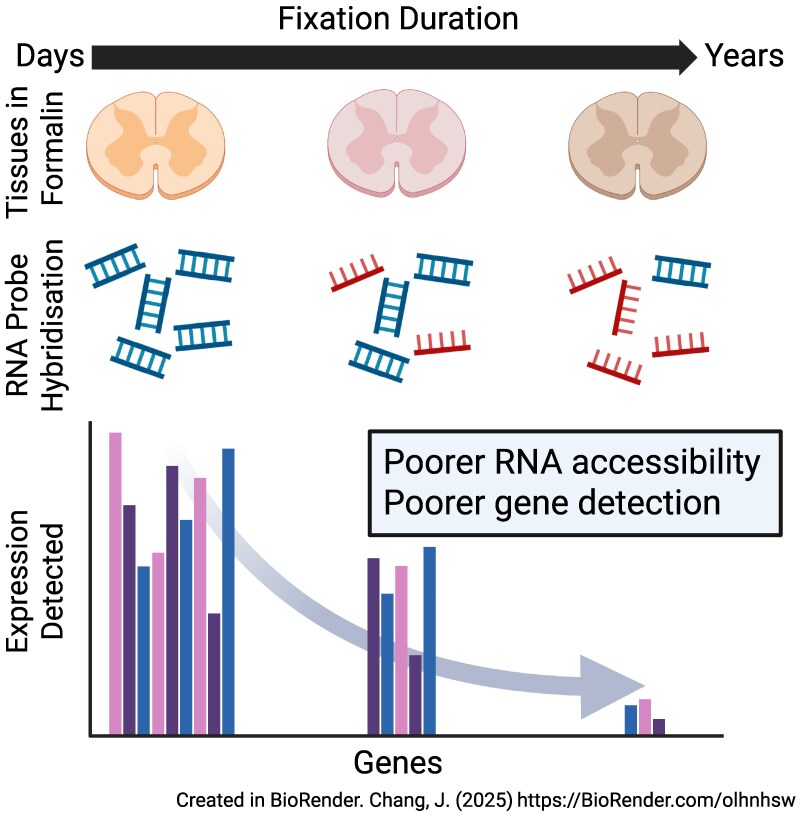

## Introduction

The degradation of donated post-mortem tissues presents a significant challenge for scientists investigating tissue morphology and protein expression. To this end, fixation techniques aim to preserve the morphology of tissues by introducing cross-links between proteins and between proteins and nucleic acids.^1^ Doing so prevents the tissue from undergoing further autolysis due to hypoxia.^2^ In the last few decades, advances in RNA technologies, such as reverse-transcription PCR, have introduced new challenges for scientists to select compatible archived tissues.^3^ This has spurred the development of now commonly used RNA quality metrics such as the DV200 and RNA integrity number (RIN).^4,5^

**Figure 1:**
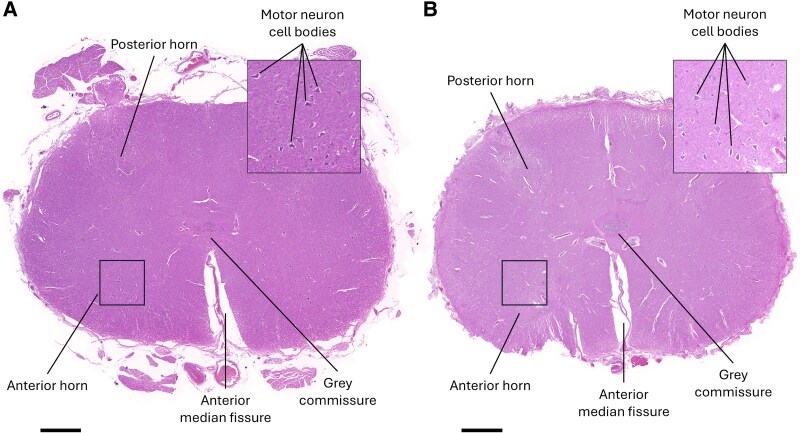
**Tissue morphology and cytoarchitecture are preserved with long-term formalin fixation.** Lumbar spinal cord sections are stained with haematoxylin and eosin. Major anatomical landmarks are labelled, and motor neuron cell bodies are indicated in the insets. Sections are from (**A**) a 2-week-fixed tissue block from a 65-year-old male donor with bvFTD-MND, and (**B**) a 6-year-fixed tissue block from a 67-year-old female donor with bvFTD. Black scale bars are 1000 μm.

More recent advances in RNA technologies have enabled greater comprehension of the transcriptome at the single-cell/nuclei and spatial level across various biological tissues,^6^ organisms^7^ and diseases.^8^ Notably, sequencing-based spatial transcriptomics technologies, a natural progression of single-cell RNA sequencing, have become popular methods of understanding transcriptome expression in a spatial context.^9^ Brain banks are an important source of human brain tissue specimens that enable investigations of human neurological diseases that can only be conducted post-mortem.^10^ As a result, transcriptomics technologies have been developed to be compatible with archived tissue formats such as formalin-fixed paraffin-embedded (FFPE) blocks.^11^ Time in fixative can, however, vary greatly within and between brain banks, ranging from weeks to years.^12^ While fixation preserves tissue morphology and cytoarchitecture, even after prolonged periods (Fig. 1), the process introduces chemical modifications to proteins and nucleic acids.^13^ The effect of fixation time for FFPE tissues has been investigated previously for imaging-based transcriptomics technologies, suggesting a negative impact of fixation on RNA detection.^14^

In this study, we aim to assess the impact of fixation duration for FFPE tissues for sequencing-based transcriptomics technologies—Chromium Next Gem Flex and Visium (10x Genomics). We report a significant and negative impact of extended fixation times on the capture of RNA, severely impacting the interpretability of the transcriptome. As similar technologies become more widely used, our results suggest a need for careful selection of tissues, including consideration of fixation duration, prior to attempting next-generation sequencing-based spatial transcriptomics studies. Moreover, this also highlights the need for brain banks to consider alternative tissue collection or post-processing protocols and subsequent standardization of these protocols across banks in order to provide optimal samples for emerging technologies such as spatial and single-nuclei transcriptomics.

## Materials and methods

### Donors and study design

Donor tissues were obtained from the Sydney Brain Bank at Neuroscience Research Australia from 2010 to 2022. Hypothalamic and lumbar spinal cord tissues originated from four donors diagnosed clinically with motor neuron disease (MND), five donors with a diagnosis of behavioural variant frontotemporal dementia (bvFTD), and four donors with a diagnosis of bvFTD-MND. All donors had confirmed pathogenic repeat expansions in the *C9ORF72* gene and confirmed TDP-43 pathology and neuronal loss consistent with either MND and/or bvFTD. A full list of donors and fixation times is presented in Supplementary Table 1. Each donor contributed tissue sections sampled from the hypothalamus and/or spinal cord. Each tissue section was subjected to different experimental conditions, including varying fixation times and RNA technologies. In our analysis, we accounted for the donor of origin for each sample using linear mixed models (see Statistical analysis).

Brain and spinal cord tissues were dissected within an hour of tissue retrieval, and samples of strategic regions were collected. For each donor, half brains and spinal cords were fixed for approximately 2 weeks in 15% neutral buffered formalin before the cerebrum was sliced coronally and the spinal cord sliced transversely. Three-millimetre-thick samples from strategic brain regions were processed and embedded in paraffin as previously described,^10^ and representative mid-regions of cervical, thoracic, lumbar and sacral spinal cords were paraffin-embedded.

Remaining fixed tissues were placed in 10% formalin for long-term storage. Formalin-fixed hypothalamus samples had longer fixation times, as the hypothalamus was not initially designated as a priority region during early processing. All paraffin blocks and tissues in 10% formalin were stored at room temperature. Unfixed half brain slices and sampled regions were stored at −80°C; once frozen, they were transferred to labelled plastic bags for long-term storage. For this project, these fresh frozen hypothalamic tissues were placed in 10% formalin for 2 days, then processed and embedded in paraffin. Full processing and storage conditions for each sample are provided in Supplementary Table 1. Hypothalamic tissues fixed for 2 days were obtained from three donors with MND, three donors with bvFTD and two donors with bvFTD-MND. Hypothalamic tissues in fixative for longer than 6 years were obtained from two donors with bvFTD and two with bvFTD-MND. All hypothalamic tissues were processed for single-nuclei sequencing.

Lumbar spinal cord tissues from four donors with MND and three donors with bvFTD-MND were in fixative for 2 weeks. Of these, all spinal cord tissues were processed for single-nuclei sequencing. Three MND tissue sections and three bvFTD-MND tissue sections were processed for spatial transcriptomics. Additionally, lumbar spinal cord tissues from three donors with bvFTD and one with bvFTD-MND were in fixative for longer than 6 years. Of these, two bvFTD tissue sections were processed for single-nuclei sequencing, and all tissue sections were processed for spatial transcriptomics.

### RNA quality testing

To test for RNA quality metrics, DV200 and RIN, RNA was extracted from samples using the Qiagen RNeasy FFPE Kit (cat no. 73504). Extracted RNA was sent to a core sequencing facility for RNA quality testing using the Agilent Bioanalyzer or RNA ScreenTape assay. RNA quality metrics could not be attained for one lumbar spinal cord tissue from an MND donor and hypothalamic tissues from two bvFTD donors and two bvFTD-MND donors.

### Library construction and sequencing

For single-nuclei transcriptomics, tissue sections were deparaffinized and dissociated using the 10x Genomics demonstrated protocol (CG000632 rev D). For the dissociation step, we opted to use the gentleMacs Octo Dissociator. Library construction was conducted according to the Chromium fixed RNA profiling user guide (CG000527 rev F). Paired-end dual-index (read 1: 28 bp, i7 index: 10 bp, i5 index: 10 bp, read 2: 90 bp) sequencing was conducted on the NovaSeq 6000 platform (Illumina). The raw sequencing base-call data was demultiplexed using Bcl2Fastq (v2.20). Raw data were processed using Cell Ranger (v7.2.0).

For spatial transcriptomics, the 10x Genomics Visium CytAssist tissue preparation guide was followed (CG000518 rev D). FFPE blocks were sectioned at 5 μm onto VWR SuperFrost Plus Micro Slides, then shipped and stored with silica desiccant until deparaffinization, decrosslinking and imaging was performed according to the Visium CytAssist spatial gene expression for FFPE demonstrated protocol (CG000520 rev C). Libraries were constructed according to the Visium CytAssist spatial gene expression user guide (CG000495 rev F). Paired-end dual-index (read 1: 28 bp, i7 index: 10 bp, i5 index: 10 bp, read 2: 50 bp) were sequenced using either an SP100 or S1 kit on the NovaSeq 6000 (Illumina). Raw data were processed using Space Ranger (v3.0.1).

### Statistical analysis

Data for each sample were imported with Seurat (v5.3.0)^15^ in the R software environment (v4.4.0).^16^ Due to the severe sparsity of data for over-fixed samples, very generous filtering thresholds were used for initial processing. For single-nuclei samples, nuclei with fewer than two or more than 2500 genes expressed were removed from further analysis. For Visium samples, spots with less than 100 genes expressed were removed. Nuclei or spots with more than 10% mitochondrial genes were also filtered from further analysis. All samples were then log-normalized. To calculate the principal components, the 600 most variable genes were identified, and the count matrices were scaled and centred. We chose 600 variable genes because some samples had low numbers of genes with non-zero variance. However, principal components could not be calculated for four over-fixed single-nucleus samples and one spatial sample due to the extreme sparsity of their count matrices.

Shared nearest neighbour graphs were constructed using the Annoy algorithm with the Euclidean metric applied to the first 50 principal components, with *k* set to 20. To visualize samples in two dimensions, uniform manifold approximation and projection (UMAP) was conducted using the first 50 principal components with default Seurat parameters. Clusters were identified using the Leiden algorithm at a resolution of 0.5.^17^ Cluster-specific marker genes were identified using the Wilcoxon rank sum test and were considered only if they were expressed in at least 10% of nuclei or spots in either group. Genes were deemed differentially expressed if the log_2_ fold change exceeded 0.1 and adjusted *P*-values were less than 0.05. The top 50 marker genes for each cluster are listed in Supplementary Table 2. Each cluster was then annotated with a cell type. For single-nuclei datasets, this indicates the cell type identity for each nucleus, while for spatial datasets, this represents the dominant cell type under each spot. Annotations required at least three established genes per cluster (Supplementary Table 3), with clusters lacking sufficient marker genes classified as ‘Unknown’. To distinguish clusters classified as the same cell type within the same sample, representative marker genes were appended to their labels.

Wilcoxon rank-sum tests with continuity correction were conducted to compare single nuclei samples between 2 and 14 days of fixation. Brown-Forsythe tests were conducted to test for equality of group variances. To assess the long-term impact of fixation, we employed linear mixed-effects models on all samples. Technology, anatomical region and disease type were treated as fixed effects, while donor identity was included as random effects to account for inter-donor biological variability. Models were estimated using restricted maximum likelihood with the nloptwrap optimiser for parameter estimation (lme4 package v1.1-37).^18^ The *P*-values were calculated using a Wald t-distribution (report package v0.6.1).^19^  *P*-values were adjusted with the false discovery rate. Multivariate analysis of variance was used to analyse group demographic differences for continuous variables. Fisher’s exact test was used for categorical variables.

### Ethical statement

This study was approved by the University of Queensland (2021/HE001798). The Sydney Brain Bank holds ethical approval through the University of New South Wales to collect, characterise, store and distribute brain and spinal cord samples from prospectively consented donors (HC200026).

## Results

### Demographics and fixation duration

A total of 30 tissue samples were processed for analysis, including 12 hypothalamic sections and eight lumbar spinal cord sections for single-nuclei sequencing and 10 lumbar spinal cord sections for spatial transcriptomics. A summary of donor demographics is shown in Table 1. Age of death (*P* = 0.67), sex (*P* = 0.48), postmortem delay (*P* = 0.38) and diagnosis (*P* = 0.89) are balanced across tissue and technology conditions. Fixation duration is not balanced between conditions (*P* < 0.01) due to the availability of tissues.

**Table 1:** Demographic comparison between experimental groups

	Single-nuclei		Spatial	*P*
	Hypothalamus (*n* = 12)	Spinal cord (*n* = 8)	Spinal cord (*n* = 10)	
Age at death	66.33 (3.75)	64.88 (4.61)	66.40 (3.69)	0.67
Sex (female)	8	3	5	0.48
Post-mortem delay (hours)	21.75 (8.56)	28.88 (13.22)	26.6 (13.16)	0.38
Diagnosis				
MND	3	4	3	
bvFTD	5	2	3	
bvFTD-MND	4	2	4	0.89
Fixation duration				
2 days	8	0	0	
14 days	0	6	6	
>6 years	4	2	4	<0.01

Values for continuous variables presented as mean (standard deviation). Values of categorical values presented as counts. Multivariate analysis of variance was used for assessing differences between continuous variables and Fisher's exact test is used for categorical variables.

### Quality metrics

**Figure 2:**
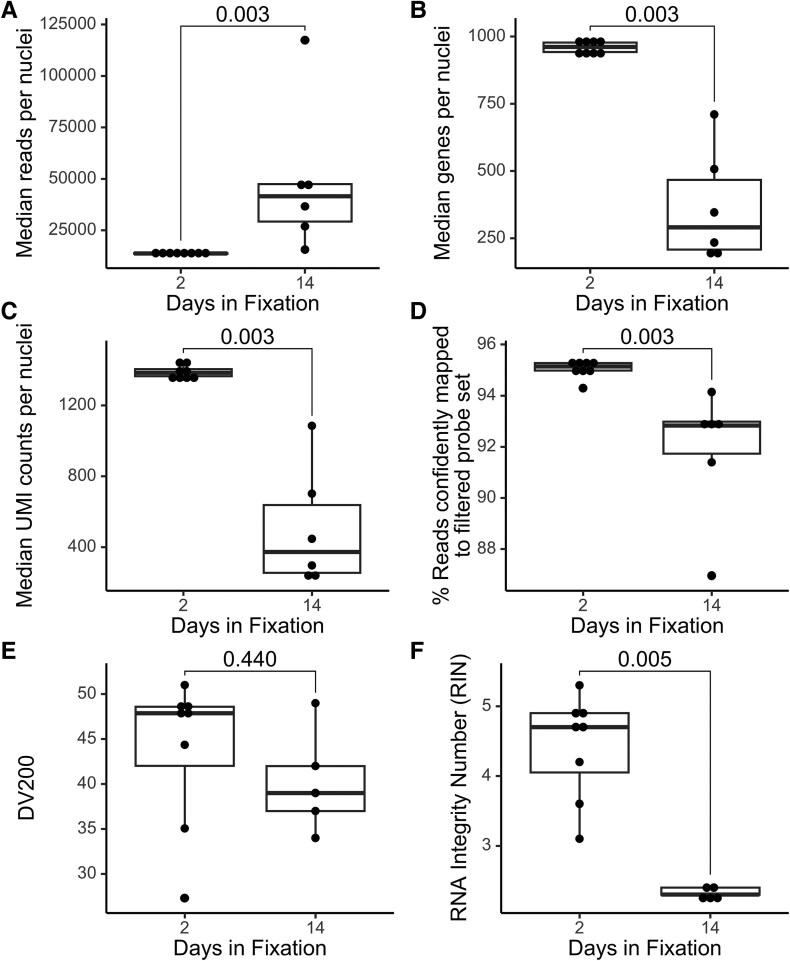
**Plots of transcriptomic and RNA quality metrics per nuclei between 2 and 14 days of fixation.** Metrics shown are (**A**) central measure of reads per nuclei, *t*(22) = −1.19, *P* = 0.431; (**B**) median number of genes per nuclei, *W* = 0, *P* = 0.003; (**C**) median UMI counts per nuclei, *W* = 48, *P* = 0.003; (**D**) % reads confidently mapped to filtered probe set, *W* = 48, *P* = 0.003; (**E**) DV200, *W* = 26, *P* = 0.440 and (**F**) RIN, *W* = 40, *P* = 0.005. Each dot represents an independent observation, with data across all experimental conditions originating from *N* = 10 donors. Tests are conducted using Wilcoxon rank-sum tests with continuity correction and *P*-values are adjusted by false discovery rate.

Comparisons of quality metrics reveal significant differences between single nuclei samples with 2 and 14 days of fixation (Fig. 2). Number of reads per nuclei are significantly greater at 14 days compared to 2 days of fixation (Fig. 2A, *P* = 0.003), while number of genes per nuclei (Fig. 2B, *P* = 0.003), median UMIs per nuclei (Fig. 2C, *P* = 0.003) and percentage of reads confidently mapped to the human transcriptome probe set (Fig. 2D, *P* = 0.003) are significantly lower at 14 days than 2 days of fixation. DV200 (Fig. 2E, *P* = 0.440) is not different between the two groups, and RIN (Fig. 2F, *P* = 0.005) is significantly lower at 14 days compared to 2 days of fixation. Tests for equality of group variances trended to significance for number of reads (*P* = 0.085), genes (*P* = 0.085) and UMI counts per nuclei (*P* = 0.085) but were not significant for percentage confidently mapped reads (*P* = 0.163), DV200 (*P* = 0.730) and RIN (*P* = 0.150).

**Figure 3:**
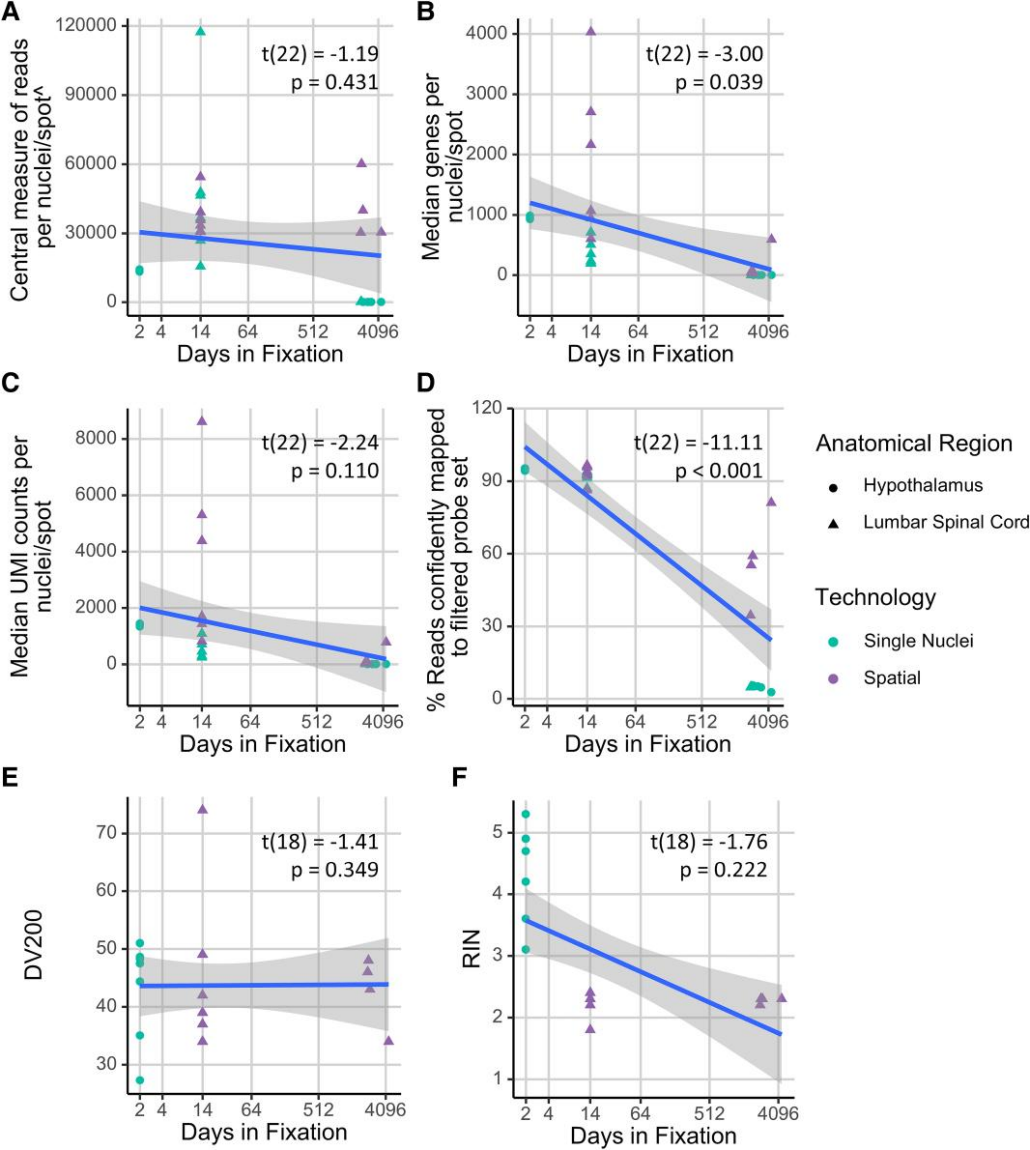
**Plots of transcriptomic and RNA quality metrics per nuclei (single-nuclei) or spot (spatial) as a function of days in fixation.** Metrics shown are (**A**) central measure of reads per nuclei/spot, *t*(22) = −1.19, *P* = 0.431; (**B**) median number of genes per nuclei/spot, *t*(22) = −3.00, *P* = 0.039; (**C**) median UMI counts per nuclei/spot, *t*(22) = −2.24, *P* = 0.110; (**D**) % reads confidently mapped to filtered probe set, *t*(22) = −11.11, *P* = 4.78 × 10^−9^; (**E**) DV200, *t*(18) = −1.41, *P* = 0.349 and (**F**) RNA integrity number (RIN), *t*(18) = −1.76, *P* = 0.222. Each dot represents an independent observation, with data across all experimental conditions originating from *N* = 13 donors. Linear mixed-effects models were used, with donors treated as random effects, and technology, anatomical region and disease treated as fixed effects. The *P*-values indicate significance with respect to days in fixation and are adjusted by false discovery rate. The *x*-axis is log(2) transformed to improve readability of graphs. ^Next Gem uses median and Visium uses mean.

We next examined the impact of long-term fixation (Fig. 3). For this analysis, nuclei and spots are treated as the same unit of measure. We observe a significant effect of fixation duration on the median number of genes per nucleus or spot (Fig. 3B, *P* = 0.039). The percentage of reads confidently mapped to the human transcriptome probe set is also highly associated with fixation length (Fig. 3D, *P* < 0.001). On the other hand, fixation duration is not associated with the number of reads per nuclei or spot (*P* = 0.431), UMIs per nuclei or spot (Fig. 3C, *P* = 0.110), DV200 (Fig. 3E, *P* = 0.349) and RIN (Fig. 3F, *P* = 0.222).

Details of other fixed effects are shown in Supplementary Table 4. Visium has a significantly higher number of median genes (*P* = 0.027) and UMI (*P* = 0.039) per spot, and a higher percentage of reads mapped (*P* = 0.039), due to its larger capture area. Anatomical region also has a significant fixed effect on the number of reads per nuclei or spot (*P* = 0.049) and RIN (*P* < 0.001).

### Clustering and cell-type annotation

Unique molecular identifier count matrices were filtered to ensure data quality for downstream analysis. The drop in the number of nuclei or spots with fixation length is significant (*P* = 0.002). Hypothalamic nuclei drop from an average of 2348 ± 223.62 nuclei at 2 days of fixation to 69.25 ± 65.10 nuclei for hypothalamic samples in fixative for over 2000 days. Similarly, spinal cord nuclei drop from an average of 366.8 ± 264.66 at 14 days of fixation to 34.50 ± 26.26 nuclei for spinal cord samples in fixative for over 2000 days. Finally, the number of spinal cord spatial spots retained after filtering dropped from 6531 ± 1192.01 spots at 14 days of fixation to 2206 ± 2343.85 spots for spinal cord samples in fixative for over 2000 days.

**Figure 4:**
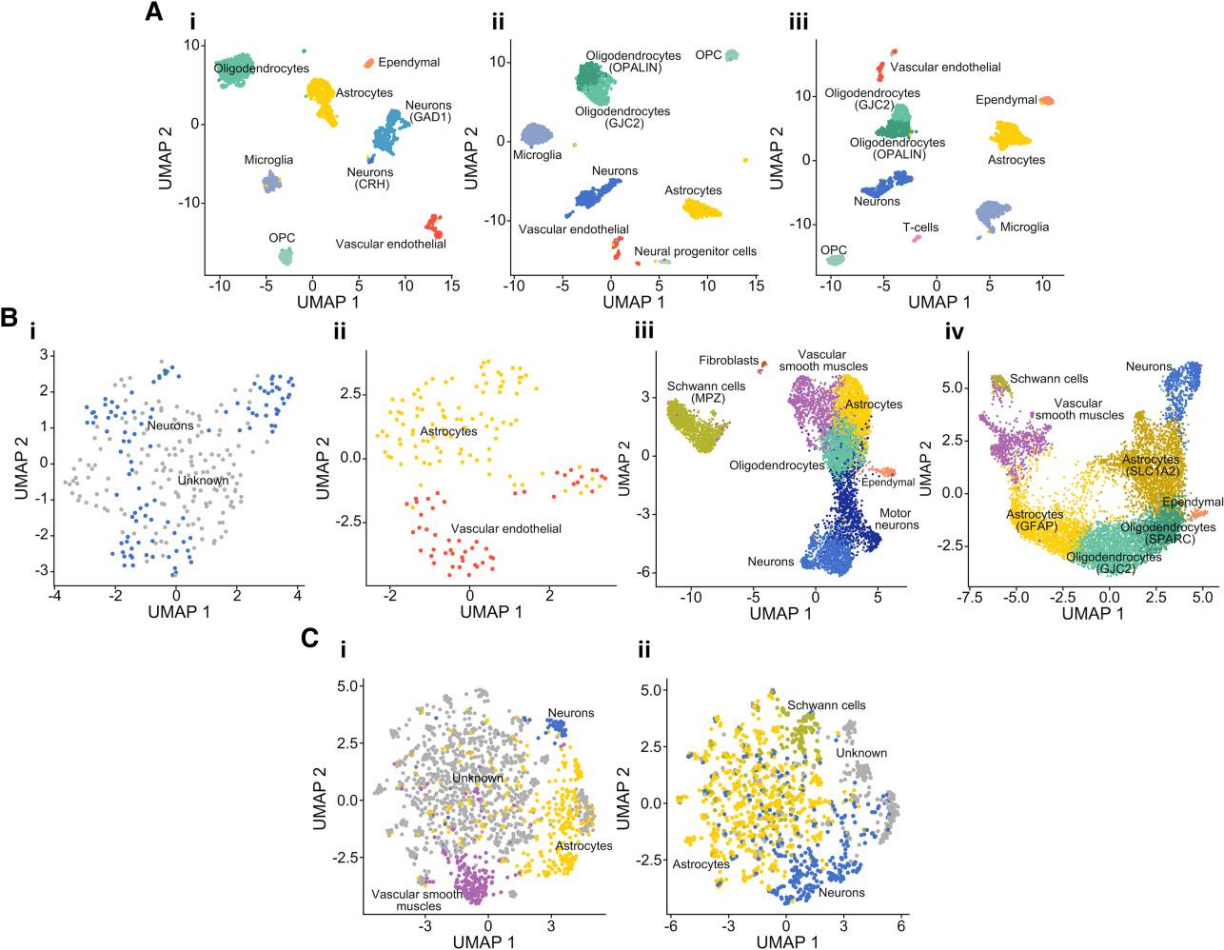
**Visualization of transcriptional profiles of samples as two-dimensional UMAP plots.** Clusters are annotated with predicted cell types using known marker genes (Supplementary Table 4). (**A**) Hypothalamus single nuclei samples with 2-day fixations from donors with (i) bvFTD, (ii) bvFTD-MND and (iii) MND. (**B**) Spinal cord (i–ii) single-nuclei and (iii–iv) spatial transcriptomics samples with 14-day fixations from donors with (i, iii) bvFTD-MND and (ii, iv) MND. (**C**) Spinal cord (i-ii) spatial transcriptomics samples with over 6 years of fixation from donors with bvFTD.

To understand the collective effect of fixation time on the whole transcriptome, we clustered and annotated the samples using standard steps in Seurat’s single-cell analysis pipeline.^15^ Common hypothalamic cell types were identified across the bvFTD-MND disease spectrum in the 2-day fixed single-nuclei samples (Fig. 4A). Identified cell types include neurons, oligodendrocytes, oligodendrocyte precursor cells, astrocytes, microglia, ependymal motile ciliated cells and vascular endothelial cells. However, analysis of 14-day-fixed single-nuclei samples reveals fewer clusters (two clusters versus seven to nine clusters) and cell types (Fig. 4B(i-ii)).

In contrast, spinal cord spatial transcriptomics samples retain informative clusters at 14 days of fixation (Fig. 4B(iii-iv)). Dominant cell types identified under transcriptomic spots across these samples include Schwann cells, vascular smooth muscle cells, astrocytes, oligodendrocytes, ependymal ciliated motile cells and neurons. Of note, motor neurons were identified in bvFTD-MND (Fig. 4B(iii)) but not in MND (Fig. 4B(iv)).

After more than 6 years of fixation, however, very little transcriptomic biological information is retained. No UMAPs could be generated for hypothalamic samples due to the severe sparsity of gene expression data. While spinal cord spatial transcriptomics data could still be visualized as UMAPs, fewer clusters and cell types could be identified (Fig. 4C).

### Principal components and marker gene expressions

**Figure 5:**
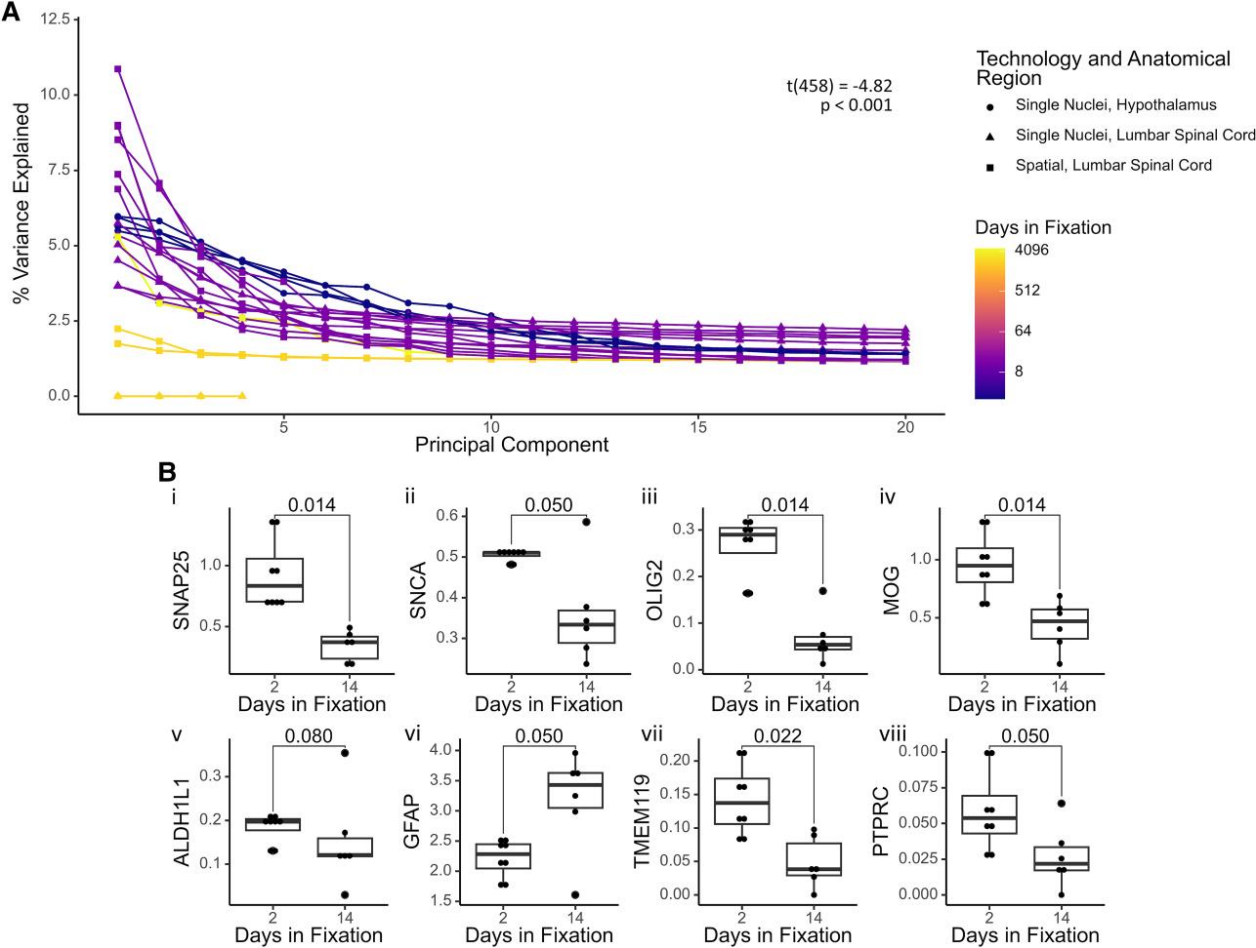
**Expression and interpretability of transcriptome and marker genes.** (**A**) % variance explained up to 20 principal components; *t*(458) = −4.82, *P* = 1.96 × 10^−6^. Principal component analysis was unable to be performed on five samples due to the sparsity of gene expression. Each line represents an independent observation, with data across all experimental conditions originating from *N* = 13 donors. Data are modelled using linear mixed effects with donors treated as random effects, and technology, anatomical region and disease treated as fixed effects. (**B**) Average expression of key marker genes between two and 14 days of fixation. Marker genes shown are (i) *SNAP25*, *W* = 48, *P* = 0.014; (ii) *SNCA*, *W* = 40, *P* = 0.050; (iii) *OLIG2*, *W* = 46, *P* = 0.014; (iv) *MOG*, *W* = 46, *P* = 0.014; (v) *ALDH1L1*, *W* = 38, *P* = 0.080; (vi) *GFAP*, *W* = 8, *P* = 0.050; (vii) *TMEM119*, *W* = 44, *P* = 0.022 and (viii) *PTPRC*, *W* = 40, *P* = 0.050. Each dot represents an independent observation, with data across all experimental conditions originating from *N* = 10 donors. Comparisons are conducted using Wilcoxon rank-sum tests with continuity correction and *P*-values are adjusted by false discovery rate.

To understand how the principal components contribute to the variations in visualization and clustering, we analysed each sample’s elbow plots of the first 20 principal components (Fig. 5A). Results indicate a significant effect of fixation duration on the variance explained by the principal components (*P* < 0.001). Variance explained is also significantly associated with the technology and anatomical region (*P* = 0.005).

We next investigated the difference in average gene expression per nucleus between 2 and 14 days of fixation (Fig. 5B). Marker genes for neurons (*SNAP25* and *SNCA*), oligodendrocytes (*OLIG2* and *MOG*), astrocytes (*ALDH1L1* and *GFAP*) and microglia (*TMEM119* and *PTPRC*) were chosen for analysis. We observe significant decreases in gene expression for *SNAP25* (*P* = 0.014), *SNCA* (*P* = 0.050), *OLIG2* (*P* = 0.014), *MOG* (*P* = 0.014), *ALDH1L1* (*P* = 0.080), *TMEM119* (*P* = 0.022) and *PTPRC* (*P* = 0.050). Curiously, average *GFAP* expression is increased at 14 days of fixation (*P* = 0.050). Finally, we note there are no significant differences in group variances for any of these genes.

We next looked at the long-term impacts of fixation on gene expression (Supplementary Fig. 1). We observe significantly negative effects of fixation duration on the expression for *SNAP25* (*P* < 0.001), *SNCA* (*P* < 0.001), *OLIG2* (*P* < 0.001), *MOG* (*P* < 0.001), *ALDH1L1* (*P* = 0.004), *GFAP* (*P* < 0.001), *TMEM119* (*P* = 0.002) and *PTPRC* (*P* < 0.001).

Details of fixed effects are shown in Supplementary Table 5. Visium spots capture higher expression compared to single nuclei: *SNAP25* (*P* = 0.005), *SNCA* (*P* < 0.001), *OLIG2* (*P* < 0.001), *ALDH1L1* (*P* < 0.001), *GFAP* (*P* < 0.001) and *TMEM119* (*P* = 0.006). Interestingly, anatomical region has a significant effect on *GFAP* expression (*P* = 0.004).

## Discussion

Tissue fixation is an integral step in the preparation of tissues for many life-science applications, including transcriptomics. How long-term fixation affects RNA for sequencing-based transcriptomics technologies is still unknown. We conducted Chromium Next Gem Flex and Visium (10x Genomics) experiments on 30 human post-mortem hypothalamic and lumbar spinal cord samples. We demonstrate the significant impacts of long-term fixation on RNA for these sequencing-based transcriptomics technologies and the interpretability of the resulting transcriptome. When considering traditional measures of RNA quality, no changes were detected with long-term fixation duration. However, when considering post-library construction measures, we see significant associations of fixation times with gene counts and reads mapped to the transcriptome. Moreover, principal component and cluster analyses indicate significant impacts of fixation time on biological variance and interpretability of the transcriptome. As transcriptomics studies on post-mortem human tissues become more widespread, these findings provide critical confirmation of the importance of compatible human tissue processing procedures.

The aim of formaldehyde fixation is to ‘freeze’ biological molecules in time.^20^ This leads to the introduction of protein–nucleic acid cross-links,^13^ addition of mono-methylol (hydroxymethyl) groups on nucleic bases, methylene bridges between adenine bases,^21^ and fragmentation of the RNA—with 200 nucleotides cited as the top limit of RNA fragment size in FFPE tissues.^22^ DV200 was developed in 2014 to determine the percentage of RNA fragments exceeding 200 nucleotides, a relevant metric for assays that use reverse transcription PCR amplification.^4^ On the other hand, RIN was developed in the early 2000s as an algorithm-based measure that considers the distribution of total RNA fragments through electrophoretic separation.^5^ In both technologies used in our investigation, DV200 is recommended by the manufacturer as the sole RNA quality metric for determining RNA integrity in FFPE samples. In our analysis, we observe drops in RIN from 2 to 14 days of fixation. However, we do not observe an association of long-term fixation with either DV200 or RIN. As these measures are dependent on RNA fragment size, our results suggest extended fixation times do not measurably impact the fragmentation of RNA. Others similarly report limited effects of formalin fixation on measures of RNA quality and RNA fragmentation itself for fixation lengths in the first few hours and days.^23-25^ One report found substantial effects of formalin fixation on RIN in the first 12 h.^26^ The dynamic range for RIN is likely to extend over several days and weeks, whereas DV200 primarily reflects changes occurring within only the first few hours of fixation. Consequently, these measures on samples subjected to prolonged fixation may not be informative. Given brain banks often fix tissues for weeks or years, it would be reasonable to assume that DV200 and RIN provide limited value for the determination of RNA quality in FFPE archival tissues where fixation length is highly variable.

The number of detected reads per transcriptional unit initially increases at 14 days of fixation, then remains stable with longer-term fixation; however, we note this is a measure primarily influenced by sequencing depth. By contrast, significant drops are observed in the fraction of reads confidently mapped to the human transcriptome probes. This is an indication of single probes that are unligated, non-specific binding of reads or pairing with probes that are mismatched. The increase in non-specific probe binding with fixation time is also observed in imaging-based transcriptomics.^14^ Correspondingly, the number of genes significantly decreases with fixation length. Thus, while the length and quantity of RNA molecules remain steady with fixation time, their biochemical structure may be severely affected, preventing the proper access and ligation of transcriptome probes to their target sites. This is not unexpected, considering that prolonged fixation leads to excessive and irreversible cross-links and biochemical changes to RNA molecules.^1^ Additionally, the minimal impact on UMIs in the longer term suggests that fixation could disproportionately affect certain genes more than others.

Lower rates of mapped transcriptome probes have a direct impact on the sparseness of the resulting gene expression matrix, affecting the interpretability of the transcriptome. The decrease in the number of genes and filtered nuclei or spots amounts to the loss of data. This is exemplified through visualization of clusters and cell types in the UMAPs (Fig. 3). The identified cell types in 2-day-fixed hypothalamic samples closely align with the heterogeneous cell types identified in the reference human hypothalamic transcriptomics dataset, the HYPOMAP.^6^ While the hypothalamus is known to be involved across the MND-bvFTD disease spectrum, understanding of cellular-level pathology in these diseases remains an active area of research.^27,28^ The use of transcriptomics technology, however, appears to have a measurable impact on the interpretability of results at 14 days of fixation. Although cluster diversity and cellular annotation are hampered in the spinal cord single-nuclei samples, spatial transcriptomics samples provide informative clustering that reflects biology and disease. We demonstrate in the spinal cord the absence of a lower motor neuron cluster and increased glial cells in MND compared to bvFTD-MND. This observation aligns with the known pathologies of these conditions, where lower motor neuron dysfunction manifests to a lesser extent in bvFTD than in MND.^29^ Finally, when considering spatial transcriptomics of spinal cords fixed for over 6 years, little biological information is retained, with poor separation of clusters and up to half of the clusters not able to be identified.

These differences in analytical outcomes are reflected in the reductions in variance captured by the principal components and expression of key central nervous system marker genes with fixation time. Though, this is not a perfect correlation, and other factors like fixation quality, post-mortem delay and storage time may contribute to these reductions. Practically, this means less abundant cell types and lowly-expressed genes are more difficult to detect in over-fixed archived tissues (formalin fixed for more than 48 h). Additionally, each gene is affected differently, with some genes, like *GFAP*, showing increased detection, possibly due to subtle differences in the chemical properties of transcriptome probes. This is supported by previous findings showing that gene-specific RNA transcript properties impact their detection.^30^ Another possibility is the influence of the log normalization step, which may inflate highly expressed genes (i.e. *GFAP*) in samples with low total gene counts. A disproportionate change in GFAP is notable as it may artificially inflate astrocytic signatures in over-fixed tissues, potentially introducing bias into the interpretations of glial–neuronal interactions in transcriptomics studies. Overall, the collective impact on the transcriptome may obfuscate relevant biological and disease pathways. The use of over-fixed archived tissues for transcriptomics experiments represents a technical challenge and can impede knowledge gain and understanding of specific disease mechanisms.

Evident in the linear mixed effects modelling and the cluster analysis, Visium assays on 2-week-fixed tissues may still provide informative results. This is not surprising, given Visium probes are intentionally designed to bind to fragmented RNA, and transcriptomic spots are 55 µm, capturing several cells. As such, some over-fixed tissues may still provide biologically informative insights at the supracellular level. However, spatial transcriptomics technologies with supracellular resolution are often complemented by single-cell or single-nuclei RNA sequencing, enabling bioinformatics methods like label transfer and cell-type deconvolution.^31^ Such techniques may be hindered by the greater loss of data in single-nuclei RNA sequencing that originates from over-fixed tissues.

Anatomical region also has some significant impacts on results; however, in the case of our samples, we cannot disentangle the region with differences in the initial preparation of the tissue blocks, post-mortem. This is especially the case for the hypothalamus, which is not a commonly investigated area of the human brain. This is a limitation of the present study, and one that future research can address by working more closely with tissue banks, from initial collection of tissues to ensure all variables are controlled and procedures are optimized for transcriptomics experiments. Another limitation is the limited fixation timepoints in the first few hours, days and between 14 and 2000 days. Such data would strengthen the effect size and be informative to determine the optimal fixation time for transcriptomics experiments. Similarly, our results suggest fixation lengths should not exceed 2 days. Experiments were also conducted in multiple batches; however, we did not account for this due to the limited sample size. Finally, we note fixation length is not balanced between transcriptomics conditions. We modelled this as part of our linear mixed models, but future in-depth studies should consider more balanced study designs.

Archived human tissues from brain and tissue banks provide researchers with a wide range of opportunities for studying rare neurodegenerative diseases. However, the compatibility of tissues for assays is not guaranteed, as novel sequencing-based transcriptomics technologies emerge at a rapid pace. As is often the case, tissues are placed in fixative for extended periods of time. Collectively, we demonstrate fixation times greater than 48 h have a detrimental effect on RNA accessibility and detectability for sequencing-based transcriptomics, leading to sparser data and poorer analytical outcomes.

### Future directions

Contemporary RNA methods for FFPE tissues use probe-based approaches, circumventing the need for intact transcripts and poly(A) tails. DV200 and RIN only assess RNA fragment sizes and not their retrievability. We posit that bulk RNA exome capture may be a cost-effective and practical solution to identify suitable FFPE tissues before conducting more expensive downstream applications like spatial transcriptomics and single-nuclei RNA sequencing. Similar to the methods discussed in this manuscript, RNA exome capture uses probe-based targeting to capture known polynucleotide transcript sequences. Metrics arising from this assay, including the number of gene-mapped reads, detected genes and sample-wise correlations, may provide useful insights into the efficacy of spatial transcriptomics and single-nuclei RNA sequencing for FFPE tissues. Importantly, this approach allows researchers to interrogate genes of interest before proceeding to more costly experiments. However, validation in future studies is needed to determine its suitability as a potential quality assurance assay.

For several reasons, most brain banks perform whole- or half-organ formalin fixation for a minimum of 2 weeks, followed by strategic blocking of regions of interest. These include limited storage space for large amounts of archived tissue per case and limited staffing levels to process and maintain the materials generated. As such, consideration should be given to alternate strategies for improving researcher access to appropriate tissue. These strategies may include the short-term fixation and paraffin embedding of archival fresh frozen tissue and working closely with researchers to determine whether suitable tissues may be collected prospectively for a particular project. Communication of updated protocols across brain banks is also encouraged via existing and future brain bank networks, biobanking conferences and publications. Results demonstrate a critical need to consider fixation time as a factor for transcriptomics experiments, ensuring tissue samples provide sufficient quantities of accessible RNA for library construction.

## Supplementary Material

fcaf428_Supplementary_Data

## Data Availability

Custom scripts used to generate figures and statistical inferences for this manuscript are available at https://doi.org/10.5281/zenodo.16741561. The data that support the findings of this study are available from the corresponding author, upon reasonable request.
