# Supplementary material for: Long-term fixation impact on archived human nervous tissues for sequencing-based transcriptomics
Source: Brain Commun. 2025 Oct 30;7(6):fcaf428. doi: 10.1093/braincomms/fcaf428 (PMC12624389; doi:10.1093/braincomms/fcaf428)
Supplement: fcaf428_Supplementary_Data [file fcaf428_supplementary_data.pdf]

Supplementary Table 1 List of donor demographics, tissues and fixation times

ID	Age at death	Sex	Postmortem delay (hours)	Case	Technology	Tissue type	Fixation time (days)
1	57	Female	31	MND	Chromium Next Gem Flex	Hypothalamus	2
1	57	Female	31	MND	Chromium Next Gem Flex	Lumbar Spinal Cord	14
1	57	Female	31	MND	Visium	Lumbar Spinal Cord	14
2	59	Female	20	MND	Chromium Next Gem Flex	Lumbar Spinal Cord	14
3	65	Male	23	MND	Chromium Next Gem Flex	Hypothalamus	2
3	65	Male	23	MND	Chromium Next Gem Flex	Lumbar Spinal Cord	14
3	65	Male	23	MND	Visium	Lumbar Spinal Cord	14
4	67	Male	26	MND	Chromium Next Gem Flex	Hypothalamus	2
4	67	Male	26	MND	Chromium Next Gem Flex	Lumbar Spinal Cord	14
4	67	Male	26	MND	Visium	Lumbar Spinal Cord	14
5	65	Male	57	bvFTD-MND	Chromium Next Gem Flex	Lumbar Spinal Cord	14
5	65	Male	57	bvFTD-MND	Visium	Lumbar Spinal Cord	14
6	67	Female	22	bvFTD-MND	Chromium Next Gem Flex	Hypothalamus	4508
6	67	Female	22	bvFTD-MND	Visium	Lumbar Spinal Cord	4508
7	68	Female	9	bvFTD-MND	Chromium Next Gem Flex	Hypothalamus	2
7	68	Female	9	bvFTD-MND	Visium	Lumbar Spinal Cord	14
7	68	Female	9	bvFTD-MND	Chromium Next Gem Flex	Hypothalamus	2940
8	70	Male	15	bvFTD-MND	Chromium Next Gem Flex	Hypothalamus	2
8	70	Male	15	bvFTD-MND	Chromium Next Gem Flex	Lumbar Spinal Cord	14
8	70	Male	15	bvFTD-MND	Visium	Lumbar Spinal Cord	14
9	61	Male	39	bvFTD	Chromium Next Gem Flex	Hypothalamus	2
10	67	Female	22	bvFTD	Chromium Next Gem Flex	Hypothalamus	2
10	67	Female	22	bvFTD	Chromium Next Gem Flex	Lumbar Spinal Cord	2352
10	67	Female	22	bvFTD	Visium	Lumbar Spinal Cord	2352
11	68	Female	17	bvFTD	Chromium Next Gem Flex	Hypothalamus	3276
12	69	Female	24	bvFTD	Chromium Next Gem Flex	Hypothalamus	2
12	69	Female	24	bvFTD	Chromium Next Gem Flex	Hypothalamus	2520
12	69	Female	24	bvFTD	Visium	Lumbar Spinal Cord	2520
13	69	Male	37	bvFTD	Chromium Next Gem Flex	Lumbar Spinal Cord	2408
13	69	Male	37	bvFTD	Visium	Lumbar Spinal Cord	2408

Supplementary Table 2 Cluster marker genes

Id/tech/tissue/days fixed	Figure panel	Cluster	Annotation	Top 50 differentially expressed genes
**9/single-nuclei/hypothalamus/2**	4Ai	1	Oligodendrocytes	MOG, ENPP2, CLDN11, TF, TMEM144, CARNS1, CNDP1, SCD, CNP, PLP1, ABCA2, MBP, EDIL3, KLK6, MYRF, DBNDD2, CLDND1, SPP1, GPR37, CERCAM, RNASE1, PCSK6, MOBP, CRYAB, QDPR, PLEKHH1, UGT8, SELENOP, ERMN, APLP1, CNTN2, LPAR1, SLC44A1, EVI2A, TMEM63A, FA2H, MAL, SEPTIN4, ANLN, RAPGEF5, ELOVL1, LDB3, PPP1R14A, TTYH2, SLAIN1, MAN2A1, CDK18, SUN2, HSPA2, CAPN3
**9/single-nuclei/hypothalamus/2**	4Ai	2	Astrocytes	GJA1, EFEMP1, SLC14A1, AQP4, FGFR3, ATP1A2, TNC, AGT, CLU, MLC1, ETNPPL, ATP1B2, ATP13A4, F3, SERPINA3, CPE, APOE, FAM189A2, SDC4, ID4, ITGB4, RGMA, AEBP1, PHYHD1, PLPP3, CD44, HSPB8, GFAP, AHCYL1, SRPX, LRIG1, AQP1, DTNA, SPARCL1, PLTP, FXYD1, ITM2C, ENTPD2, PSD2, NTRK2, GRAMD2B, WLS, SERPINE2, APLNR, SLC7A11, PFKFB3, NTSR2, SORBS1, SLC1A3, PAPLN
**9/single-nuclei/hypothalamus/2**	4Ai	3	Neurons (GAD+)	SNAP25, NSG2, TMEM130, SCG2, GAD2, SYT1, NSG1, PEG3, PHYHIP, THY1, SLC32A1, SYP, NSF, ATP1A3, NCDN, RTN1, GRIN1, PTPRN, CAMKV, CHGB, NRGN, CALY, STMN2, SYN1, UCHL1, SYN2, VAT1L, RAB3A, PEG10, L1CAM, ELMOD1, SYT5, NAPB, SCN3B, ST8SIA3, GRIA1, CHN1, PACSIN1, HPCA, PRKCG, PDE1B, DNM1, STXBP1, GABRG2, LAMP5, BEX1, PREPL, SV2C, PNMA2, NDRG4
**9/single-nuclei/hypothalamus/2**	4Ai	4	Microglia	CSF1R, C1QB, LAPTM5, C1QC, CX3CR1, C1QA, GPR34, CYBB, PLD4, SELPLG, ADAM28, P2RY12, CSF2RA, ITGB2, FGL2, RGS1, LAIR1, FCGR3A, CD68, C3AR1, MS4A6A, CD4, FCER1G, LPCAT2, TREM2, CTSS, TYROBP, P2RY13, CD74, CLEC7A, RNASET2, SLC2A5, SPI1, A2M, HAVCR2, LPAR6, CD14, SERPINA1, C3, ITGAX, OLR1, FCGBP, OTULINL, APBB1IP, TMEM119, AIF1, CYBA, HCLS1, CD53, SRGN
**9/single-nuclei/hypothalamus/2**	4Ai	5	OPC	PDGFRA, TNR, MEGF11, GPR17, CSPG4, LHFPL3, SULF2, PCDH15, SMOC1, EMILIN3, DLL3, PTPRZ1, CSPG5, VCAN, BCAN, OLIG2, IGSF21, OLIG1, C1QL1, CA10, PHLDA1, LUZP2, MMP16, MYT1, EPN2, COL9A1, C1QL2, APOD, THBS4, LRRN1, KLRC3, FABP7, CHAD, PTN, PLPPR1, DSCAM, IL1RAP, WSCD1, LINGO1, ASCL1, TNK2, TMEM100, SSTR1, SCD5, SEMA5A, COL20A1, AFAP1L2, SNX22, SCG3, CACNG4
**9/single-nuclei/hypothalamus/2**	4Ai	6	Vascular endothelial	ESAM, DCN, FLT1, EPAS1, ABCG2, CDH5, VWF, CD34, MYL9, PODXL, TM4SF1, LRRC32, MMRN2, ADGRF5, ABCB1, NDUFA4L2, IFI27, CAVIN2, TINAGL1, ICAM2, SLC38A5, TGM2, RGS5, GNG11, ACTA2, ITGA1, PTPRB, FN1, ADGRL4, HIGD1B, ENG, KLF2, COBLL1, APOLD1, TAGLN, GPR4, LIMS2, SRARP, ANXA3, NOSTRIN, SLC38A11, TM4SF18, CLDN5, SEMA3G, NOTCH3, ARHGAP29, FOXC1, LSR, ITIH5, GJA4
**9/single-nuclei/hypothalamus/2**	4Ai	7	Ependymal	FAM183A, CFAP157, FAM216B, STOML3, DYDC2, LRRC71, ODF3B, FAM92B, MYLK3, ROPN1L, C11orf88, MAPK15, LRRC46, CFAP73, DNAH9, ZMYND10, CFAP52, C9orf24, DTHD1, SPATA18, PIFO, CAPSL, ANKRD66, FOXJ1, C1orf194, RSPH1, CD36, DNAH12, DYNLRB2, DNAAF3, EFCAB1, AC013470.2, PRR29, DNAI1, CCDC153, DAW1, RASSF9, TTC29, TCTEX1D1, CFAP300, EFCAB10, AKAP14, CFAP43, SPAG17, MUSK, CFAP57, MORN5, CCDC33, CFAP299, ARMC4

**9/single-nuclei/hypothalamus/2**	4Ai	8	Neurons (CRH)	SLC17A6, CPNE9, CHRNA2, KCNC2, SHOX2, PVALB, RGS16, FAM163A, CBLN2, TRBC2, HAPLN4, GALNT9, RGS8, NTNG1, NEFH, EPN3, ADRA1D, TAFA4, SHISAL1, CRH, SNCG, RASSF3, ADCYAP1, NEFM, WNT3, PTPN3, PPP1R17, CPNE7, NEFL, CACNA1G, CCDC136, MATN3, ADRA1B, DIRAS1, GNG13, SPTSSB, CHGA, SCN1B, TCF7L2, SLC24A3, C11orf87, SDR16C5, ZMAT4, CPLX1, ZDHHC22, SLC6A17, AKAIN1, RIMS3, GLRA3, OPRM1
**8/single-nuclei/hypothalamus/2**	4Aii	1	Oligodendrocytes (OPALIN)	OPALIN, QDPR, CNDP1, CNP, ENPP2, RNASE1, MAL, TMEM144, MOG, CERCAM, PLP1, SEPTIN4, ABCA2, DBNDD2, MYRF, MBP, CLDN11, EDIL3, LPAR1, SLC44A1, GPR37, TF, SUN2, CLDND1, CSRP1, HHIP, APLP1, SCD, ERMN, CD9, APOD, SLAIN1, CRYAB, HSPA2, GPRC5B, LAMP2, SELENOP, CA2, CNTN2, CARNS1, PPP1R14A, EVI2A, UGT8, MOBP, PLEKHH1, PTGDS, CAPN3, TTLL7, PMP22, KLK6
**8/single-nuclei/hypothalamus/2**	4Aii	2	Neurons	SNAP25, NSG1, TMEM130, NSG2, ATP1A3, SCG2, STMN2, UCHL1, PEG3, PRKAR1B, CALY, EEF1A2, SYT1, PNMA2, SYP, SYN1, TUBB2A, NEFL, NEFM, THY1, INA, GAD2, RTN1, TMEM59L, SYT4, PTPRN, RAB3A, PCSK1N, NSF, GRIN1, PEG10, VAT1L, BEX1, SYNGR3, GAP43, NCDN, CTXN1, ACHE, L1CAM, RAB3C, CHGB, NRGN, DNM1, NAP1L5, GNG3, KIF5A, MAP1B, SYT5, SCN3B, SYT13
**8/single-nuclei/hypothalamus/2**	4Aii	3	Oligodendrocytes (GJC2)	DHCR24, TF, ADAMTS4, MOG, KLK6, PCSK6, CARNS1, PLP1, ENPP2, FA2H, SCD, CLDN11, CLDND1, UGT8, ABCA2, MOBP, SELENOP, APLP1, EDIL3, CRYAB, ELOVL1, CNP, TMEM63A, HAPLN2, TMEM144, CNTN2, MARCKSL1, SLC44A1, RAPGEF5, FGFR2, CNDP1, GJC2, HSPA2, CERCAM, ERMN, DBNDD2, SEPTIN8, CDK18, MYRF, MBP, DPYSL5, CNTNAP4, GJB1, GPR37, TTYH2, RNASE1, PLEKHH1, ANLN, LDB3, LRP2
**8/single-nuclei/hypothalamus/2**	4Aii	4	Microglia	LAPTM5, CSF1R, C1QB, C3, CD74, C1QC, P2RY12, CX3CR1, CD68, C1QA, SELPLG, GPR34, CYBB, PLD4, TREM2, TYROBP, A2M, ADAM28, RNASET2, CD4, ITGAX, C3AR1, FCER1G, OLR1, CYBA, SPI1, AIF1, CSF2RA, FGL2, SLCO2B1, ITGB2, EHBP1L1, P2RY13, LAIR1, CD14, FXYD5, LPAR6, CD53, SRGN, KCTD12, CPVL, ALOX5AP, CXCL16, HAVCR2, CD37, APBB1IP, MS4A7, CTSS, CTSB, LY86
**8/single-nuclei/hypothalamus/2**	4Aii	5	Astrocytes	GJA1, AGT, MLC1, AQP4, TNC, EFEMP1, FGFR3, SLC14A1, AQP1, ITGB4, RGMA, PLTP, F3, ENTPD2, ATP1A2, PHYHD1, ID4, SRPX, ETNPPL, AEBP1, SDC4, CLU, CD44, FAM189A2, ATP1B2, HSPB8, SPARCL1, CPE, NTRK2, ATP13A4, PSD2, KCNN3, PAMR1, WLS, SERPINE2, EDNRB, SPON1, TRPM3, MASP1, SLC7A11, CD38, AHCYL1, DCLK2, FRMPD2, GFAP, APLNR, SORBS1, NSMF, DTNA, ABI3BP
**8/single-nuclei/hypothalamus/2**	4Aii	6	OPC	PDGFRA, GPR17, CSPG4, MEGF11, LHFPL3, TNR, SULF2, PCDH15, CHAD, COL20A1, C1QL2, CACNG4, LRRN1, VCAN, PHLDA1, PTPRZ1, CSPG5, COL9A1, MMP16, BCAN, DSCAM, SLC35F1, SEMA5A, IGSF21, C1QL1, B3GNT7, MYT1, CA10, NEU4, NXPH1, ASCL1, SEZ6L, LUZP2, FERMT1, XYLT1, OLIG1, OLIG2, CSMD1, EPN2, OLFM2, SPRY4, THBS4, CD82, PLPPR1, PCDHGC3, SUSD5, PLPP4, NRXN1, LRP1, SNX22
**8/single-nuclei/hypothalamus/2**	4Aii	7	Vascular endothelial	ESAM, TINAGL1, ABCG2, EPAS1, FOXC1, FN1, ADGRF5, HIGD1B, CAVIN2, FLT1, FHL5, ITIH5, CDH5, LRRC32, ABCB1, CFH, IFI27, NDUFA4L2, VWF, DCN, RGS5, ARHGAP29, MYO1B, CLDN5, TM4SF1, PODXL, TGM2, CD34, MYL9, IFITM3, MUSTN1, SLC38A5, PTPRB, GNG11, GPER1, COBLL1, ADGRL4, SOX18, NOTCH3, KLF2, ICAM2, LAMC3, CYYR1, EMCN, OMD, PLAT, ITGA1, TIE1, NOSTRIN, IGFBP7

**8/single-nuclei/hypothalamus/2**	4Aii	8	Neural progenitor cells	WIF1, SFTA3, RAX, TBX3, TRDN, CRYM, EDN3, AGTR1, SLC27A6, COL1A2, PTPRT, FRZB, MOXD1, ISLR, LGR6, IGFBP3, IGFBP6, FAM20A, NPTX2, ANGPTL1, ADGRV1, CYSLTR2, ADGRL2, COL25A1, FZD5, NKX2-1, MIA, NGFR, PDE5A, CHST9, KLHDC8A, LHX2, IL13RA2, MFAP2, UNC5D, LAMA2, AVPR1A, ABCA13, RDH10, STON1, PRELP, ADAMTSL1, GPX3, PDGFRL, GRIK3, CALCRL, IGFBP2, TIMP1, ECRG4, NXPH3
**4/single-nuclei/hypothalamus/2**	4Aiii	1	Astrocytes	SERPINA3, AQP4, F3, AQP1, GJA1, SDC4, CD44, AGT, APLNR, SPARCL1, CLU, GFAP, ATP1A2, EFEMP1, AEBP1, ATP1B2, SLC14A1, TNC, FGFR3, MLC1, HSPB8, ITGB4, DTNA, NTRK2, CPE, SCARA3, RGMA, ANGPTL4, AHCYL1, ETNPPL, SLC4A4, ADM, PLPP3, ATP13A4, LRIG1, WLS, EMP1, FXYD1, SLC7A11, HILPDA, SORBS1, ID4, MGST1, EDNRB, SRPX, LFNG, ENTPD2, APOE, FAM189A2, ITM2C
**4/single-nuclei/hypothalamus/2**	4Aiii	2	Microglia	CSF1R, C1QB, RGS1, C1QC, LAPTM5, C1QA, CD74, CYBB, CD14, CYBA, C3, FCGR3A, CD68, GPR34, SRGN, FGL2, CX3CR1, ADAM28, FCER1G, A2M, SLC11A1, ITGB2, CSF2RA, TYROBP, SERPINA1, ITGAX, PLD4, TREM2, P2RY12, LAIR1, SLCO2B1, AIF1, IFI30, FPR1, C3AR1, SPI1, EHBP1L1, SLC2A5, LPAR6, CTSB, SELPLG, LPCAT2, CD53, RHBDF2, ALOX5AP, RNASET2, DENND3, CD4, OLR1, MS4A6A
**4/single-nuclei/hypothalamus/2**	4Aiii	3	Oligodendrocytes (OPALIN)	TMEM144, PLP1, OPALIN, MOG, RNASE1, CNP, ABCA2, ENPP2, CLDN11, TF, MBP, CERCAM, QDPR, MAL, DBNDD2, SCD, EDIL3, MYRF, SEPTIN4, SLC44A1, ERMN, APLP1, CARNS1, GPR37, LPAR1, MOBP, SELENOP, CSRP1, LAMP2, TTYH2, CNDP1, CLDND1, PIP4K2A, CRYAB, SLAIN1, PLEKHH1, CNTN2, KCNMB4, SLCO1A2, PPP1R14A, GPM6B, APOD, SUN2, TTLL7, PHLDB1, GPRC5B, HSPA2, PTGDS, FA2H, AMER2
**4/single-nuclei/hypothalamus/2**	4Aiii	4	Neurons	TMEM130, NSG1, NSG2, SNAP25, SYT1, GRIN1, GAD2, ATP1A3, SCG2, PEG3, PTPRN, CALY, EEF1A2, SLC32A1, THY1, PEG10, PHYHIP, CAMKV, SYT5, PCSK1N, SYP, STMN2, PACSIN1, UCHL1, RAB3A, BEX1, RTN1, NCDN, ST8SIA3, L1CAM, NSF, SYN1, PRKAR1B, DNM1, CHGB, PNMA2, SCN3B, RAB3C, NEFL, ELMOD1, GABRG2, TSPYL2, ENO2, GNG3, VSTM2A, RBP4, CHN1, NDRG4, VSNL1, SYN2
**4/single-nuclei/hypothalamus/2**	4Aiii	5	Oligodendrocytes (GJC2)	ENPP6, KLK6, PCSK6, ADAMTS4, CARNS1, HAPLN2, PDIA2, UGT8, TF, MOG, GPIHBP1, CLDND1, FA2H, ENPP2, CDK18, CNTNAP4, CNDP1, LDB3, DHCR24, CNTN2, ELOVL1, SLC45A3, SLC5A11, CLDN11, GJB1, PLEKHH1, LRP2, SLC31A2, TMEM144, SELENOP, RAPGEF5, GJC2, PLP1, TTYH2, HHATL, MYRF, ABCA2, MOBP, ANLN, SCD, ERMN, HCN2, CERCAM, APLP1, TMEM63A, RNASE1, CNP, TMEM151A, DPYSL5, SYNJ2
**4/single-nuclei/hypothalamus/2**	4Aiii	6	OPC	PDGFRA, CSPG4, TNR, MEGF11, LHFPL3, GPR17, EMILIN3, CSPG5, CHAD, VCAN, BCAN, COL20A1, PTPRZ1, MMP16, B3GNT7, LRRN1, PCDH15, DLL3, SLC35F1, C1QL1, CA10, MYT1, SULF2, DSCAM, OLIG2, C1QL2, ASCL1, SMOC1, SEMA5A, TRAF4, OLIG1, EPN2, NEU4, CACNG4, THBS4, SUSD5, PLPPR1, BCHE, CALCRL, NRXN1, APOD, COL9A1, PLPP4, LUZP2, PLAT, IL1RAP, WSCD1, NXPH1, PHLDA1, IGSF21
**4/single-nuclei/hypothalamus/2**	4Aiii	7	Vascular endothelial	ESAM, DCN, TINAGL1, LRRC32, COL4A1, NDUFA4L2, FOXC1, FN1, COL4A2, NOTCH3, RGS5, GNG11, MYO1B, MYL9, CDH5, APOLD1, CFH, EPAS1, NID1, HIGD1B, ADGRF5, PDGFRB, COL3A1, TGM2, GJC1, IGFBP4, ABCG2, TM4SF1, ABCB1, LAMC3, SLC38A11, GPER1, ADGRL4, COL1A2, ARHGAP29, GGT5, FLT1, GPR4, ICAM2, MMRN2, CAVIN2, ITGA10, NOSTRIN, PHLDB2, CD248, ECM1, CCN2, GJA4, SOX18, TPM2

**4/single-nuclei/hypothalamus/2**	4Aiii	8	Ependymal	CFAP73, FAM216B, FAM183A, STOML3, MYLK3, C9orf24, RSPH1, CAPSL, LRRC46, CFAP157, C11orf88, MAPK15, FAM92B, LRRC71, EFCAB1, DTHD1, SLC47A2, CFAP52, ROPN1L, DYDC2, DAW1, RASSF9, TTC29, DYNLRB2, PRR29, C9orf116, DNAAF3, CD24, CFAP45, ODF3B, C1orf194, DNAH9, PPP1R32, ZMYND10, CFAP126, PIFO, CCDC65, C5orf49, DRC3, FOLR1, C9orf135, C4orf47, ARMC3, TEKT1, MORN5, IQCG, FOXJ1, DNAH12, EFCAB10, CD36
**4/single-nuclei/hypothalamus/2**	4Aiii	9	T-cells	CCL5, CD3E, CD8A, CD96, TRBC2, GZMA, CD2, TRAC, PRF1, CD3D, IL2RG, GZMK, CD52, CTSW, CST7, KLRB1, KLRK1, GZMH, CD7, KLRD1, CCL4, IL7R, NKG7, CD3G, IL2RB, CCR2, LCK, IL18RAP, CXCR6, IL32, TRBC1, CD69, IKZF3, SKAP1, CD247, SLAMF1, THEMIS, CD48, ZNF683, GRAP2, SLAMF6, SLAMF7, ITK, CXCR3, CYTIP, ETS1, SEPTIN1, PTGER2, GATA3, SH2D1A
**8/single-nuclei/spinal cord/2**	4Bi	1	Unknown	MBP, CRYAB, S100B, PLP1, S100A1, TF
**8/single-nuclei/spinal cord/2**	4Bi	2	Neurons	SNAP25, NEFM, NEFL, SNCG, RTN1, EEF1A2, VAMP1, DYNLL2, THY1, NPTN, NEFH, STMN2, KIF5A
**4/single-nuclei/spinal cord/2**	4Bii	1	Astrocytes	TUBB2B, CRYAB, S100B, GFAP, FEZ1, CKB, DPYSL2, DKK3, ITM2C, PMP2, MBP, AQP4, GPM6B, PADI2, MT3, DTNA, PLEKHB1, SCD, AGT, AHCYL1, S100A1, CNTN1, MARCKSL1, ALDOC, AQP1
**4/single-nuclei/spinal cord/2**	4Bii	2	Vascular endothelial	NFKBIA, EPAS1, A2M, PECAM1, TGFBR2, COL4A1, FN1, FXYD5, ENG, CLDN5, CEBPD, LIMS2, KCTD12, SRGN, CCN2, ICAM2, TM4SF1, PRSS23, DUSP1, IGFBP7, IFI27, DEPP1, VWF, COL4A2, HSPA5, CCN1, ETS2, MYL9, TIMP3, IFITM2, KLF6, MAFF, TPM4, FOXC1, CFLAR, ERO1A, VIM, JUNB, TNXB, TGM2, EHBP1L1, LGALS3, HYAL2, LMO2, IL32, TPM1, ADAMTS1, IGFBP3, ABCB1, JCAD
**8/spatial transcriptomics/spinal cord/2**	4Biii	1	Astrocytes	GFAP, PLP1, TF, PLEKHB1, SPP1, PADI2, SCD, APLNR, MBP, CRYAB, MOBP, FGFR2, APLP1, FAM107A, ERMN, MARCKSL1, PEA15, HSPA2, CERCAM, MOG, DHCR24, KLK6, AQP4, AQP1, TUBB2B, PAQR6, HSPB8, CARNS1, S100B, S100A1, SNORC, CLDND1, HAPLN2, DPYSL5, SEPTIN8, PLEKHH1, CNP, NDRG2, RASSF2, DBI, PCSK6, CDK18, SLC44A1, SLAIN1, GPRC5B, UGT8, FEZ1, CD44, GPD1, DAAM2
**8/spatial transcriptomics/spinal cord/2**	4Biii	2	Oligodendro cytes	MBP, TMSB4X, MOBP, GFAP, PLEKHB1, MT-ND4L, PAQR6, MT-ND1, MT-ND6, MTURN, MT-CYB, MT-ND4, FAM107A, MT-ATP6, SPARC, PLP1, MT-ND2, CLU, MT-CO3, AQP4, MT-ND5, APOE, HSPB8, S100A1, AQP1, MT-ND3, MARCKSL1, PEA15, MT-CO2, HSPA2, FLOT2, TF, SAFB, LUC7L, CCND1, DAZAP2, KTN1, BAG1, NEK7, RNF181, TMED7, ARAP1, MYH14, PPP2CB, TSPO, DBNL, NCBP2, NOP56, SNORC, MFF
**8/spatial transcriptomics/spinal cord/2**	4Biii	3	Schwann cells (MPZ)	PRX, DRP2, LPL, MLIP, PLEKHA4, CLDN19, RELN, MIA, AHR, MYL9, AZGP1, MME, SCN7A, CTNNAL1, COL1A2, SMTN, IGFBP6, LGI4, SFRP5, GAS2L3, LTBP4, NR4A1, SAMHD1, GNG2, LIMS2, DCN, FN1, OAF, KCNK12, COL3A1, DAG1, COL28A1, LAMB1, USP53, NRXN1, ANXA2, MPZ, CAV2, DMD, CLIC4, ITGA6, AFAP1L2, SOX10, MAL, DCLK3, ISLR, COL14A1, COL4A2, GPX3, FBLN2
**8/spatial transcriptomics/spinal cord/2**	4Biii	4	Motor neurons	NEFL, NEFM, NEFH, SNAP25, PRPH, APOE, EEF1A2, PVALB, STMN2, SLC18A3, SNCG, UTS2, ATP1B2, QDPR, CKB, ALDOC, CHODL, NCAN, MT-CO3, UCHL1, YWHAG, KCNJ16, CHI3L1, MT-CO2, SLC1A2, ARHGAP36, NDRG4, CABP7, VAMP1, CEND1, SLC6A11, MT-ND1, THY1, MT-ND5, SLC5A7, MT-CYB, CLU, C1orf61, RAB3A, SPP1, TMSB4X, MT-ATP6, ENO2, FXYD7, CRABP1, SCD5, CHAT, GNG3, SLC10A4, AQP4

**8/spatial transcriptomics/spinal cord/2**	4Biii	5	Neurons	SNAP25, STMN2, EEF1A2, NEFL, NEFM, ATP1A3, VSNL1, SNCB, RAB3A, CAMK2N2, THY1, ENO2, SNCG, SCG2, PEG3, YWHAG, TMEM59L, CEND1, NAP1L5, PACSIN1, PRKAR1B, STX1B, NCDN, GNG3, CHGB, GRIN1, PNMA8B, UCHL1, NSG2, NSF, LAMP5, SYP, CALY, PTPRN, INA, PENK, TAGLN3, CPLX2, NAPB, TMEM130, CHGA, MLLT11, PCP4, DNM1, ACHE, CALB2, PCSK1N, RTN1, NSG1, MAP2
**8/spatial transcriptomics/spinal cord/2**	4Biii	6	Vascular smooth muscles	TAGLN, ACTA2, MYH11, TPM2, JUNB, VIM, CNN1, APOD, PTGDS, KLF2, MYL9, DUSP1, NR4A1, DES, PLN, CXCL2, ADAMTS1, TPM1, CALD1, FBLN1, ZFP36, NOTCH3, DCN, CCN1, HSPB1, FHL5, CLDN5, TNXB, LMOD1, JUN, EPAS1, PTGIS, ISLR, HBA2, ACTG2, CRIP1, FHL2, FOS, FLNA, COL6A2, C11orf96, TGM2, RHOB, HSPA1B, ITGA8, IGFBP7, BGN, ID3, TINAGL1, FRZB
**8/spatial transcriptomics/spinal cord/2**	4Biii	7	Ependymal	CFAP157, C9orf24, CFAP45, MYLK3, ADGB, FAM216B, FAM183A, CFAP73, ARMC3, DNAI1, MAPK15, LRRC71, DCDC2, CAPSL, TEKT1, SPAG6, DNAAF1, C11orf88, CFAP99, ZMYND10, CCDC33, DYDC2, SLC47A2, C1orf87, LRRC46, CCDC60, SPEF1, FAM92B, CCDC153, DTHD1, DRC3, HYDIN, TCTEX1D1, CFAP52, VWA3A, C4orf47, ANKRD66, EFCAB1, CFAP77, AK7, TTC29, C6orf118, C1orf194, CFAP100, ROPN1L, C5orf49, CFAP43, FOXJ1, CFAP47, CFAP54
**8/spatial transcriptomics/spinal cord/2**	4Biii	8	Fibroblasts	C19orf33, NOTUM, LPAR3, SLPI, ASPN, KDR, ZIC2, PRDM6, DSP, FAM180A, FAM20A, ADAMTSL3, AOX1, WNK4, COL13A1, RBP4, TNFRSF11B, TMEM100, NDUFA4L2, IL1R1, EPHA7, THSD4, APOA1, SCARA5, NPNT, ZNF385B, FIBIN, ZIC5, DMKN, MFAP5, SHISAL2B, SERPIND1, DNASE1L3, FOXC2, TNNT2, CEMIP, KLF5, PKP2, GJB6, PRRX1, HIST1H2BG, MFAP2, FGF18, FOXC1, SIX1, IGF1, SFRP2, ITIH2, DSG2, FMOD
**4/spatial transcriptomics/spinal cord/2**	4Biv	1	Astrocytes (SLC1A2)	NEFL, CRABP1, HBA2, MT-CO3, MTURN, MT-ND6, MT-CO2, MT-CYB, CLDN5, CKB, APOE, MT-ND1, RGS5, SLC1A2, MBP, CHI3L1, SNAP25, ALDOC, MT-ND5, SLC6A11, SLC2A1, MT-ND4, ATP1A2, CST3, EDN1, NDRG2, SNCG, EPAS1, MT-ATP6, ATP1B2, STMN2, HBB, TMSB10, UTS2, MT-ND2, MT-ND3, CXCL14, NCAN, NEFH, CCN2, ITM2A, SNU13, RAC1, TM9SF3, ECH1, TAPBP, RAB18, ATP5PD, PODXL, ACOX1
**4/spatial transcriptomics/spinal cord/2**	4Biv	2	Oligodendrocytes (GJC2)	TF, PLP1, CRYAB, SPP1, APLP1, CNP, S100B, DHCR24, CLDND1, SELENOP, KLK6, MARCKSL1, SEPTIN8, CARNS1, ERMN, UGT8, ABCA2, TMEM144, SCD, CERCAM, HSPA2, EDIL3, DBNDD2, PCSK6, MOG, S100A1, DPYSL5, CLDN11, QKI, FMNL2, ENPP2, PEA15, ANLN, MYRF, FGFR2, HAPLN2, SLC44A1, PPP1R14A, STMN4, MOBP, PLEKHH1, RASSF2, GPM6B, RAPGEF5, TUBB2B, ELOVL1, FEZ1, SLAIN1, FA2H, MYLK
**4/spatial transcriptomics/spinal cord/2**	4Biv	3	Astrocytes (GFAP)	APLNR, ADM, GFAP, RGS1, DDIT4, GJA1, FLNC, CAPS, CPAMD8, SFRP2, PFKFB3, SPP1, DBI, CD99, AQP4, GPM6B, CD74, FBXO2, AEBP1, PSAP, TUBA1A, HILPDA, CHI3L2, DPYSL3, C3, CD44, METRN, CXXC5, MAOB, SBSPON, NUPR1, PLCD3, DKK3, ITGB4, GALNT15, MASP1, PPP1R1B, SNORC, DDR1, TNR, S100B, GOLM1, ASAH1, MLC1, HSPB8, MT3, VCAN, FAM107A, C1QC, SLC38A1
**4/spatial transcriptomics/spinal cord/2**	4Biv	4	Oligodendrocytes (SPARC)	MBP, MOBP, TMSB4X, MTURN, PAQR6, SPARC, FXYD6, MRFAP1L1, EIF3A, CFL2, ZFYVE21, MIF, PGRMC1, ERBIN, TOMM20, N4BP2L2, ALCAM, CAPG, RERE, MYEF2, MPV17, OGFRL1, TTC3, RSRP1, PURA, TAPBP, ARL8A, ETFB, LANCL1, STAU2, HNRNPH3, RBX1, EPS15, ALDH1A1, EEF1B2, SEC13, PSMB1, TBCB, GMPR, SAR1B, KIF5B, DCAF8, ACYP2, CRYAB, ATP6V1E1, ALAD, PDK4, HIPK1, NCL

**4/spatial transcriptomics/spinal cord/2**	4Biv	5	Vascular smooth muscles	DCN, IGFBP6, COL1A2, FBLN1, PRELP, C1S, MYL9, TNXB, GPX3, TAGLN, MGP, CXCL12, ISLR, C1R, COL6A2, FBLN5, CEBPD, TPM1, SERPING1, ZFP36, SFRP4, LTBP2, CD163, NBL1, TPM2, CEMIP, COL1A1, C7, ACTA2, PCOLCE, FBLN2, MFAP4, COL3A1, IGFBP4, FMOD, IGFBP2, OGN, TYRP1, FRZB, LYVE1, THBS1, COL6A3, MARCO, MYH11, VIM, CES1, ISM1, CYP1B1, BGN, GSN
**4/spatial transcriptomics/spinal cord/2**	4Biv	6	Neurons	SNCG, SNAP25, EEF1A2, THY1, CALY, NCDN, STMN2, ATP1A3, GNG3, RAB3A, CHGB, SCG2, NSG2, PENK, SNCB, PRKAR1B, CAMK2N2, GAP43, TMEM130, VSNL1, NAPB, LAMP5, PACSIN1, GRIN1, HOXB8, SST, AMPH, CALB1, SYT1, PTPRN, JPH4, LY6H, CHGA, BAIAP3, HPCA, RAB3C, JPH3, DNM1, CALB2, EBF3, TAC1, SYT5, VSTM2L, GNG4, INA, GAD1, PDYN, CAMKV, GABBR2, TMEM59L
**4/spatial transcriptomics/spinal cord/2**	4Biv	7	Schwann cells	LPL, PRX, MLIP, DRP2, RELN, MME, AZGP1, CLDN19, SFRP5, PLEKHA4, MIA, NCMAP, SCN7A, CDH19, LUM, GAS2L3, DCLK3, WDR86, SLIT2, FGFBP2, GPC3, IL17B, PRRG4, DHH, VIT, KRT14, FOXD3, BMP8B, CTNNAL1, SMIM41, FGF7, GALNT17, INSC, EGFL8, PRRG3, KCNK12, AURKB, MPZ, MLPH, CLDN1, OAF, IL34, CFH, BIRC7, EGR2, L1CAM, SORCS1, AFAP1L2, NGFR, SMTN
**4/spatial transcriptomics/spinal cord/2**	4Biv	8	Ependymal	CFAP157, MAPK15, DNAI1, LRRC71, FAM216B, CFAP45, DRC3, EFCAB1, CCDC114, DNAAF1, DTHD1, ROPN1L, ADGB, CFAP43, CFAP73, CAPSL, SPATA18, SPAG8, C9orf24, AK7, FAM183A, BAIAP3, SPAG6, LRRC46, RSPH1, CFAP100, ZMYND10, CFAP46, WDR63, ANKRD66, RIBC2, SPEF1, FAM92B, CD36, ZBBX, MAP3K19, DAW1, CCDC153, C6orf118, CCDC33, ODF3B, MS4A2, CFAP58, CFAP97D2, ARMC3, CCDC40, AL590560.2, ADGRD2, HYDIN, TTC6
**12/spatial transcriptomics/spinal cord/2520**	4Ci	1	Unknown	MPZ, S100B, CRYAB, PMP2, AZGP1, APOD, SPARC, SFRP5, PMP22, FGFBP2, ENO1, ITGB8
**12/spatial transcriptomics/spinal cord/2520**	4Ci	2	Astrocytes	GFAP, MBP, SPARC, APOE, CST3, AQP4, PLEKHB1, RASSF2
**12/spatial transcriptomics/spinal cord/2520**	4Ci	3	Vascular smooth muscles	TAGLN, TPM2, ACTA2, C11orf96, MYL9, CNN1, LMOD1, ADIRF, NOTCH3, HSPB1, HES4, TPM1, CALD1, COL4A2, IGFBP4, MYH11, TGM2, C1QTNF1, MYL6, MCAM, MGP, FLNA, CAVIN3, FN1, DES, COL4A1, EHD2, LPP, FBLIM1, HSPB6, CAVIN1, IGFBP5, GPX3, ADAMTS1, TNS1, PPP1R14A, JUNB, YBX3, DSTN, MRGPRF, PDK4, RHOB, VIM, KCNMB1, MUSTN1, CEBPD, ACTN4, ACTG2, IGFBP7, EHBP1L1
**12/spatial transcriptomics/spinal cord/2520**	4Ci	4	Unknown	SLC6A18, KLLN, KRT78, EPB41L1, GPR143, USP27X, KLK11, SCIMP, PPFIA2, TTBK2
**12/spatial transcriptomics/spinal cord/2520**	4Ci	5	Neurons	NEFL, STMN2, NEFM, UCHL1, EEF1A2, NEFH, CPLX2, ACOT7, SNCG, CHGA, PVALB, RAB3A, YWHAG, MLLT11, THY1, SNCB, SNAP25, C9orf16, ENO2, CAMK2N2, TUBA4A, PRPH, STMN3, NDRG4, CEND1, ATP5IF1, SV2C, ATP1A3, EHD3, EPDR1, REEP2, CALM3, MAPK9, GNG3, ACHE, STX1B, ALDOC, SYT2, TCF7, DDX58, BASP1, SYP, CALY, SYNGR3, PTPRN, SLC17A6, ADAM23, CABP1, SCAMP5, NCDN
**10/spatial transcriptomics/spinal cord/2352**	4Cii	1	Astrocytes	GFAP, CNPY3, RAP2B, GNAQ, TPM2, KTN1, GJA1, HSP90AA1, APOL6, LZTS2, HSPB1, TXN2, JMJD6, TMEM14A, HSPB8, AQP4, DNAJB1, MAFF, MKNK2, G0S2
**10/spatial transcriptomics/spinal cord/2352**	4Cii	2	Neurons	SNCG, NEFL, UCHL1, PENK, NEFM, STMN2, CARTPT, EEF1A2, SNCB, CHGB, ACOT7, PCSK1N, SCG2, RAB3A, APOE, SNAP25, SCAMP5, GAP43, BASP1, CPLX2, CAMK2N2, SST, CEND1, ATP5IF1, GNAO1, PTPRN, TUBA4A, MT-ND6, GNB5, STMN3, C9orf16, PVALB, CALM3, ENO2, YWHAG, PEG3, RTN1,

				PNMA8B, PNOC, NDRG4, NCDN, TPPP3, BEX3, CCK, LAMP5, MAP2, SLC22A17, BEX5
**10/spatial transcriptomics/spinal cord/2352**	4Cii	3	Schwann cells	MPZ, PMP22, VIM, TAX1BP3, SFRP5, AZGP1, APOD, FTH1, FGFBP2, IL17B, MYL9, FN1, AHNAK, S100A6, SMTN, LPL, COL4A2, AKR1B1, CAVIN3, MAF, S100B, SNCA, CHD4, LIMS2, BIRC7, MME, COL4A1, FGF1, CRYAB, MAPK8IP1, CCN2, PMP2, COL1A2, S100A1, SMIM41, RELN, MAL, KCTD11, FGL2, MLIP, GJB1, PRX, SCARA5, MIA, HSPA12A, GSN, CLDN19, INF2
**10/spatial transcriptomics/spinal cord/2352**	4Cii	4	Unknown	HSPB1, GFAP, SPACA6, USP21, TRIM45, TRIM33
**10/spatial transcriptomics/spinal cord/2352**	4Cii	5	Unknown	RIIAD1, CCDC138, C2CD6, SIRPB1, KRTAP13-4, KRT79, BCL7C, CXXC4, STEAP3, WNT7A, TRMT10C
**10/spatial transcriptomics/spinal cord/2352**	Cii	6	Unknown	CLDND1, PON3, CANT1, DMRT3, WEE1, HSD17B12, DMXL2, FBXO15, ZKSCAN4, SMIM14, NEDD4, KLF15, RIOX1, DCAF6, CTSE, ZNF804B, PAXIP1, PRM2, SPHKAP, NXPH1, ADGRA1

Supplementary Table 3 Cell-type marker genes for the annotation of clusters

Cell type	Key marker genes	References
Microglia	CSF1R, C1QB, C1QC, C1QA, CX3CR1, P2RY12, PTPRC, TMEM119, CD68, TREM2	1,2
Oligodendrocytes	MBP, PLP1, MOG, CNP, MYRF, MOBP	3,4
Oligodendrocyte precursor cells (OPCs)	PDGFRA, CSPG4, OLIG1, OLIG2, SOX10, NKX2-2	5
Ependymal motile ciliated cells	CFAP45, CFAP73, CFAP157, DNAI1, DCDC2, TEKT1, ZMYND10, DYDC2	6-11
Astrocytes	AQP4, GLUL, GFAP, ALDOC, ALDH1L1, APOE, S100B, S100A1, GJA1, SLC1A2	12,13
T-cells	CD3E, CD3D, TRAC, TRBC1, TRBC2, CD8A, CD69, PRF1	14-17
Schwann cells	MPZ, PMP22, SOX10, DRP2, PRX	18,19
Fibroblasts	DCN, COL1A1, COL1A2, COL3A1, FN1	20
Vascular endothelial cells	CDH5, FLT1, KDR, PECAM1, VWF, CLDN5	21-23
Vascular smooth muscle cells	MYH11, TAGLN, ACTA2, CNN1, TPM2, TPM1	24,25
Neurons	NEFL, NEFM, SNAP25, SNCA, SYP, GAD1, UCHL1	26-30
Lower motor neurons	CHAT, SLC5A7, SLC18A3, CHODL, RET	31
Neural progenitor cells	RAX, NKX2-1, TBX3, LHX2	32

Supplementary Table 4 Statistics of fixed effects in linear mixed models on quality control metrics

	**Technology (Visium)**	**Anatomical region (spinal cord)**	**Case (MND)**	**Case (bvFTD-MND)**
**Reads**	t(22) =0.55, p = 0.609	t(22) = 2.73, p = 0.049	t(22) = 0.78, p = 0.591	t(22) = 0.05, p = 0.963
**Genes**	t(22) = 3.36, p = 0.027	t(22) = -0.91, p = 0.550	t(22) = 0.67, p = 0.595	t(22) = 0.67, p = 0.595
**UMI**	t(22) = 2.98, p = 0.039	t(22) = -0.60, p = 0.595	t(22) = 0.60, p = 0.595	t(22) = 0.62, p = 0.595
**Mapped**	t(22) = 2.89, p = 0.039	t(22) = 1.99, p = 0.150	t(22) = 0.81, p = 0.591	t(22) = 0.97, p = 0.530
**DV200^**	N/A	t(18) = 1.71, p = 0.225	t(18) = -1.10, p = 0.473	t(18) = -1.25, p = 0.427
**RIN^**	N/A	t(18) = -6.71, p <0 .001	t(18) = -2.20, p = 0.116	t(18) = -2.72, p = 0.050

T statistic degrees of freedom are indicated in parentheses. P-values are adjusted by false discovery rate.
^Technology is excluded as a fixed effect as these measures were conducted before experiments.

Supplementary Table 5 Statistics of fixed effects in linear mixed models on genes of interest

	**Technology (Visium)**	**Anatomical region (spinal cord)**	**Case (MND)**	**Case (bvFTD-MND)**
SNAP25	t(22) = 3.61, p = 0.005	t(22) = -2.39, p = 0.061	t(22) = -0.09, p = 0.948	t(22) = 1.01, p = 0.497
SNCA	t(22) = 9.19, p < 0.001	t(22) = -0.31, p = 0.884	t(22) = 0.06, p = 0.954	t(22) = 2.03, p = 0.112
OLIG2	t(22) = 5.77, p < 0.001	t(22) = -2.49, p = 0.052	t(22) = -0.45, p = 0.841	t(22) = -0.46, p = 0.841
MOG	t(22) = 1.78, p = 0.166	t(22) = -1.90, p = 0.138	t(22) = -0.99, p = 0.497	t(22) = -0.81, p = 0.597
ALDH1L1	t(22) = 4.00, p = 0.002	t(22) = -0.40, p = 0.870	t(22) = 2.29, p = 0.072	t(22) = 0.30, p = 0.884
GFAP	t(22) = 12.06, p < 0.001	t(22) = 3.66, p = 0.004	t(22) = 1.31, p = 0.337	t(22) = -0.78, p = 0.602
TMEM119	t(22) = 3.48, p = 0.006	t(22) = -1.26, p = 0.352	t(22) = -0.16, p = 0.935	t(22) = 0.30, p = 0.884
PTPRC	t(22) = 1.36, p = 0.326	t(22) = -1.55, p = 0.243	t(22) = 0.10, p = 0.948	t(22) = -0.88, p = 0.560

T statistic degrees of freedom are indicated in parentheses. P-values are adjusted by false discovery rate.

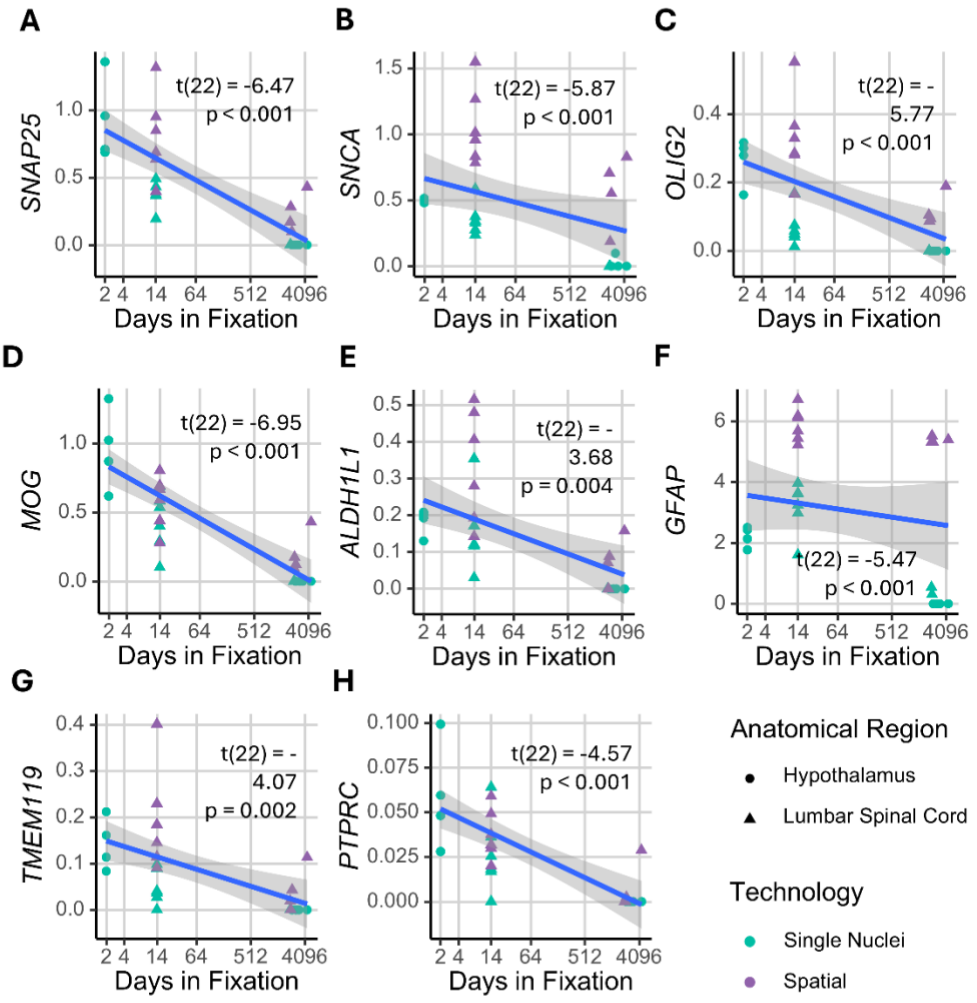

Supplementary Figure 1 Expression of key marker genes as a function of days in fixation.

Marker genes shown are (**A**) *SNAP25*, t(22)=-6.47, p=1.49×10^{-5}; (**B**) *SNCA*, t(22)=-5.87, p=4.70×10^{-5}; (**C**) *OLIG2*, t(22)=-5.77, p=4.70×10^{-6}; (**iv**) *MOG*, t(22)=-6.95, p=6.37×10^{-6}; (**v**) *ALDH1L1*, t(22)=-3.68, p=4.40×10^{-3}; (**D**) *GFAP*, t(22)=-5.47, p=8.60×10^{-5}; (**E**) *TMEM119*, t(22)=-4.07, p=2.08×10^{-3}; and (**F**) *PTPRC*, t(22)=-4.57, p=6.78×10^{-4}. Each dot represents an independent observation, with data across all experimental conditions originating from N=13 donors. The x-axis is log(2) transformed to improve readability of graphs. Linear mixed effects models were used, with donors treated as random effects, and technology, anatomical region, and disease treated as fixed effects. The p-values indicate significance with respect to days in fixation and are adjusted by false discovery rate.

References

1. Jurga AM, Paleczna M, Kuter KZ. Overview of General and Discriminating Markers of Differential Microglia Phenotypes. *Front Cell Neurosci*. 2020;14:198. doi:10.3389/fncel.2020.00198
2. Mangogna A, Belmonte B, Agostinis C, et al. Prognostic Implications of the Complement Protein C1q in Gliomas. *Front Immunol*. 2019;10:2366. doi:10.3389/fimmu.2019.02366
3. Rajkowska G, Mahajan G, Maciag D, et al. Oligodendrocyte morphometry and expression of myelin - Related mRNA in ventral prefrontal white matter in major depressive disorder. *J Psychiatr Res*. Jun 2015;65:53-62. doi:10.1016/j.jpsychires.2015.04.010
4. Koenning M, Jackson S, Hay CM, et al. Myelin gene regulatory factor is required for maintenance of myelin and mature oligodendrocyte identity in the adult CNS. *J Neurosci*. Sep 5 2012;32(36):12528-42. doi:10.1523/jneurosci.1069-12.2012
5. Ye D, Wang Q, Yang Y, et al. Identifying Genes that Affect Differentiation of Human Neural Stem Cells and Myelination of Mature Oligodendrocytes. *Cell Mol Neurobiol*. Jul 2023;43(5):2337-2358. doi:10.1007/s10571-022-01313-5
6. McCafferty CL, Papoulas O, Lee C, et al. An amino acid-resolution interactome for motile cilia identifies the structure and function of ciliopathy protein complexes. *Dev Cell*. Mar 24 2025;60(6):965-978.e3. doi:10.1016/j.devcel.2024.11.019
7. Brody SL, Pan J, Huang T, et al. Undocking of an extensive ciliary network induces proteostasis and cell fate switching resulting in severe primary ciliary dyskinesia. *Sci Transl Med*. Jan 29 2025;17(783):eadp5173. doi:10.1126/scitranslmed.adp5173
8. Mazor M, Alkrinawi S, Chalifa-Caspi V, et al. Primary ciliary dyskinesia caused by homozygous mutation in DNAL1, encoding dynein light chain 1. *Am J Hum Genet*. May 13 2011;88(5):599-607. doi:10.1016/j.ajhg.2011.03.018
9. Hoh RA, Stowe TR, Turk E, Stearns T. Transcriptional program of ciliated epithelial cells reveals new cilium and centrosome components and links to human disease. *PLoS One*. 2012;7(12):e52166. doi:10.1371/journal.pone.0052166
10. Leung MR, Sun C, Zeng J, et al. Structural diversity of axonemes across mammalian motile cilia. *Nature*. Jan 2025;637(8048):1170-1177. doi:10.1038/s41586-024-08337-5
11. Cho KJ, Noh SH, Han SM, et al. ZMYND10 stabilizes intermediate chain proteins in the cytoplasmic pre-assembly of dynein arms. *PLoS Genet*. Mar 2018;14(3):e1007316. doi:10.1371/journal.pgen.1007316
12. Jurga AM, Paleczna M, Kadluczka J, Kuter KZ. Beyond the GFAP-Astrocyte Protein Markers in the Brain. *Biomolecules*. Sep 14 2021;11(9)doi:10.3390/biom11091361
13. Zhang Y, Sloan SA, Clarke LE, et al. Purification and Characterization of Progenitor and Mature Human Astrocytes Reveals Transcriptional and Functional Differences with Mouse. *Neuron*. Jan 6 2016;89(1):37-53. doi:10.1016/j.neuron.2015.11.013
14. Lee MS, Hanspers K, Barker CS, Korn AP, McCune JM. Gene expression profiles during human CD4+ T cell differentiation. *Int Immunol*. Aug 2004;16(8):1109-24. doi:10.1093/intimm/dxh112
15. Morgan NV, Goddard S, Cardno TS, et al. Mutation in the TCRα subunit constant gene (TRAC) leads to a human immunodeficiency disorder characterized by a lack of TCRαβ+ T cells. *J Clin Invest*. Feb 2011;121(2):695-702. doi:10.1172/jci41931
16. Horna P, Weybright MJ, Ferrari M, et al. Dual T-cell constant β chain (TRBC)1 and TRBC2 staining for the identification of T-cell neoplasms by flow cytometry. *Blood Cancer J*. Feb 29 2024;14(1):34. doi:10.1038/s41408-024-01002-0
17. Wang M, Windgassen D, Papoutsakis ET. Comparative analysis of transcriptional profiling of CD3+, CD4+ and CD8+ T cells identifies novel immune response players in T-cell activation. *BMC Genomics*. May 16 2008;9:225. doi:10.1186/1471-2164-9-225
18. Svaren J, Meijer D. The molecular machinery of myelin gene transcription in Schwann cells. *Glia*. Nov 1 2008;56(14):1541-1551. doi:10.1002/glia.20767
19. Ma D, Wang B, Zawadzka M, et al. A Subpopulation of Foxj1-Expressing, Nonmyelinating Schwann Cells of the Peripheral Nervous System Contribute to Schwann Cell Remyelination in

the Central Nervous System. *J Neurosci*. Oct 24 2018;38(43):9228-9239. doi:10.1523/jneurosci.0585-18.2018
20. Rubio K, Molina-Herrera A, Pérez-González A, et al. EP300 as a Molecular Integrator of Fibrotic Transcriptional Programs. *Int J Mol Sci*. Aug 1 2023;24(15)doi:10.3390/ijms241512302
21. Lother A, Bergemann S, Deng L, Moser M, Bode C, Hein L. Cardiac Endothelial Cell Transcriptome. *Arterioscler Thromb Vasc Biol*. Mar 2018;38(3):566-574. doi:10.1161/atvbaha.117.310549
22. Shibuya M. Vascular endothelial growth factor receptor-1 (VEGFR-1/Flt-1): a dual regulator for angiogenesis. *Angiogenesis*. 2006;9(4):225-30; discussion 231. doi:10.1007/s10456-006-9055-8
23. Hashimoto Y, Greene C, Munnich A, Campbell M. The CLDN5 gene at the blood-brain barrier in health and disease. *Fluids Barriers CNS*. Mar 28 2023;20(1):22. doi:10.1186/s12987-023-00424-5
24. Khachigian LM, Black BL, Ferdinandy P, De Caterina R, Madonna R, Geng YJ. Transcriptional regulation of vascular smooth muscle cell proliferation, differentiation and senescence: Novel targets for therapy. *Vascul Pharmacol*. Oct 2022;146:107091. doi:10.1016/j.vph.2022.107091
25. Prunotto M, Bruschi M, Gunning P, et al. Stable incorporation of α-smooth muscle actin into stress fibers is dependent on specific tropomyosin isoforms. *Cytoskeleton (Hoboken)*. Jun 2015;72(6):257-67. doi:10.1002/cm.21230
26. Yuan A, Nixon RA. Neurofilament Proteins as Biomarkers to Monitor Neurological Diseases and the Efficacy of Therapies. *Front Neurosci*. 2021;15:689938. doi:10.3389/fnins.2021.689938
27. Noor A, Zahid S. A review of the role of synaptosomal-associated protein 25 (SNAP-25) in neurological disorders. *Int J Neurosci*. Sep 2017;127(9):805-811. doi:10.1080/00207454.2016.1248240
28. Delenclos M, Burgess JD, Lamprokostopoulou A, Outeiro TF, Vekrellis K, McLean PJ. Cellular models of alpha-synuclein toxicity and aggregation. *J Neurochem*. Sep 2019;150(5):566-576. doi:10.1111/jnc.14806
29. Yuan X, Li W, Yan Q, Ou Y, Long Q, Zhang P. Biomarkers of mature neuronal differentiation and related diseases. *Future Sci OA*. Dec 31 2024;10(1):2410146. doi:10.1080/20565623.2024.2410146
30. Ohtsuka N, Badurek S, Busslinger M, Benes FM, Minichiello L, Rudolph U. GABAergic neurons regulate lateral ventricular development via transcription factor Pax5. *Genesis*. Apr 2013;51(4):234-45. doi:10.1002/dvg.22370
31. Enjin A, Rabe N, Nakanishi ST, et al. Identification of novel spinal cholinergic genetic subtypes disclose Chodl and Pitx2 as markers for fast motor neurons and partition cells. *J Comp Neurol*. Jun 15 2010;518(12):2284-304. doi:10.1002/cne.22332
32. Lu F, Kar D, Gruenig N, et al. Rax is a selector gene for mediobasal hypothalamic cell types. *J Neurosci*. Jan 2 2013;33(1):259-72. doi:10.1523/jneurosci.0913-12.2013
